# Study of the Photoinduced Fate of Selected Contaminants in Surface Waters by HPLC‐HRMS

**DOI:** 10.1002/rcm.10075

**Published:** 2025-05-20

**Authors:** Rossella Sesia, Federica Dal Bello, Claudio Medana, Rita Binetti, Dimitra Papagiannaki, Paola Calza

**Affiliations:** ^1^ Department of Management and Production Engineering Politecnico di Torino Torino Italy; ^2^ Department of Molecular Biotechnology and Health Sciences University of Turin Torino Italy; ^3^ SMAT Torino Italy; ^4^ Department of Chemistry University of Torino Torino Italy

**Keywords:** coumarin, epoxiconazole, HPLC‐HRMS, hymecromone, toxicity

## Abstract

**Rationale:**

Photoinduced transformation of contaminants of emerging concern (CECs) can occur in aquatic environment and could lead to the formation of transformation products (TPs) of greater concern than the parent compounds. For such, the fate of epoxiconazole, hymecromone, and coumarin in water was investigated by simulating photoinduced abiotic transformations to assess the toxicity of their TPs and which CEC may be of greatest concern.

**Methods:**

Heterogeneous photocatalysis with TiO_2_ and direct photolysis of selected CECs were exploited to simulate their TPs. The TPs were assessed by means of HPLC coupled with an Orbitrap MS analyser in ESI positive mode, while their toxicity was evaluated through a *Vibrio fischeri* bioluminescence assay, and ECOSAR tool.

**Results:**

The formation of numerous TPs via different photoinduced pathways was noticed (27 for epoxiconazole, 6 for coumarin, and 8 for hymecromone, some of which are in the form of structural isomers). Toxicity assessment via *V. fischeri* assay showed that, unlike coumarin species, epoxiconazole transformation proceeds through the formation of toxic compounds. By means of ECOSAR software, the formation of predominant more noxious TPs of epoxiconazole was proved than the parent compound for both acute and chronic toxicities. Instead, most TPs of coumarin and hymecromone generally exhibited “harmful” and “toxic” levels of acute and chronic toxicities.

**Conclusions:**

A probable structural identification was assigned to the monitored TPs via HPLC‐HRMS to recognize the several transformation pathways, of which the hydroxylation reaction was predominant, and which compound may be more hazardous in the aquatic system due to its TPs. Epoxiconazole transformation brought to potentially toxic TPs, whereas photoinduced degradation of coumarin and hymecromone resulted in less hazardous TPs. The most significant aspect of this work is the ability of this overall approach to identify the formation of photoinduced TPs that are potentially more toxic than the original CEC.

## 1 Introduction

Contaminants of emerging concern (CECs) enter in the aquatic system through many sources, especially wastewater treatment plants, hospitals, and agriculture [1, 2, 3]. Despite their low concentration in water, CECs are potential sources of pollution [4]. Indeed, once introduced into the aquatic system, CECs are subjected to transport phenomena, leading to bioconcentration and bioaccumulation, as well as degradation processes [5]. As the range of possible pollutants to be monitored is expanding to include their transformation products (TPs), a comprehensive study about CECs transformation is multifaceted. Besides, TPs may provide a greater environmental risk than their parent species [5, 6]. Among degradation processes that CECs can undergo in surface waters, photoinduced reactions play a crucial role [7, 8]. Heterogeneous photocatalysis is among the advanced oxidation processes that allow not only achieving an efficient CECs removal but can also enable the laboratory‐based simulation of CECs photoinduced degradation [9, 10, 11]. Due to the interaction of the photocatalyst with light, reactive species are produced such as electrons, holes, and ^•^OH radicals, which can transform CECs [12, 13].

Within this framework, we investigated the photoinduced transformation of three CECs, which were selected for this study after a sampling campaign performed on lakes and river waters. Indeed, the presence of epoxiconazole and hymecromone was monitored in several samples of surface water of the Po River and Lake Orta in Italy between August and October 2020. The photodegradation of coumarin, the basic structural unit of hymecromone, was studied as well to deeply investigate the hymecromone TPs. In addition, coumarin itself may be an important plant biomarker unrelated to synthetic drugs.

**FIGURE 1:**
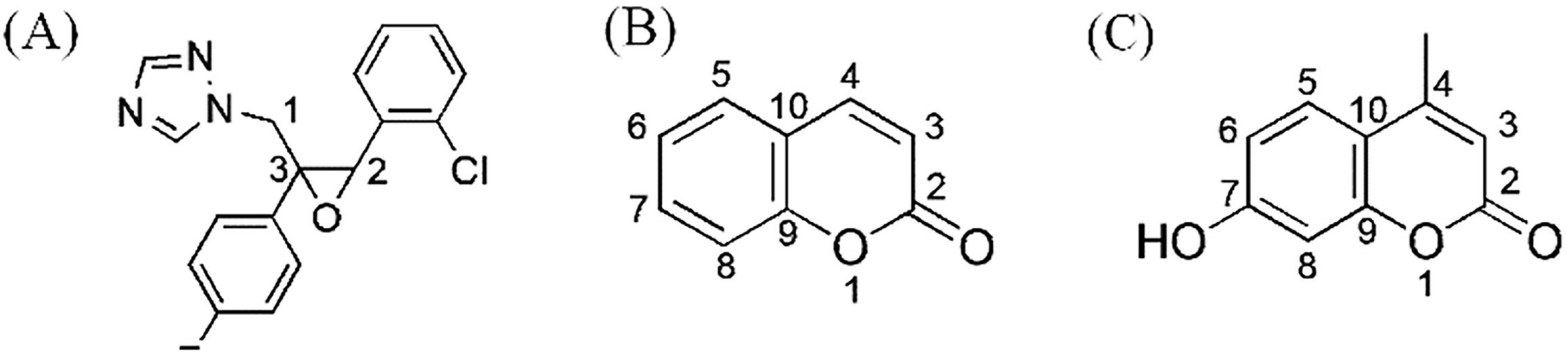
Chemical structures of epoxiconazole (A), coumarin (B), and hymecromone (C).

Epoxiconazole (Figure 1A) is a fungicide with a high environmental and human health concern due to its widespread use in agricultural field. In accordance with the Globally Harmonized System (GHS) of Classification and Labelling of Chemicals, this fungicide has been listed by the European Union as a category 2 carcinogen and having a category 1A or 1B reproductive toxicity [14]. Moreover, epoxiconazole may inhibit some enzymes of cytochrome P450, thus acting as an endocrine disruptor for nontarget organisms [15]. Finally, the extended use of epoxiconazole raises environmental concerns owing to its persistence and its presence in aquatic effluents [16, 17, 18]. Therefore, conazole fungicides have been added to the Candidate List based on the persistence, bioaccumulative, and toxic criteria [18]. Furthermore, we studied coumarin (Figure 1B) with its derivative, hymecromone (Figure 1C, 7‐hydroxy‐4‐methylcoumarin), both detected in a sampling campaign performed in rivers and effluent waters between August and October 2020. Hymecromone is used for its medicinal potentials and as a building block for the synthesis of some insecticides [18]. Environmental Protection Agency (EPA) and Canada Domestic Substances List classified hymecromone as non‐bioaccumulative, non‐intrinsically toxic to aquatic organisms, and nonpersistent [19]. However, their photoinduced fate in water has not been documented in the literature yet, and no information is available about their TPs, resulting in an incentive for their further study.

Therefore, their monitoring and the characterization of their TPs structures are the purpose of this work. Liquid chromatography (LC) is the most effective technique, as well as the high‐resolution mass spectrometry (HRMS) for the characterization of TPs through multiple‐stage analyses. The recognition of abiotic transformation pathways of epoxiconazole and the two coumarin species in the euphotic zone of surface waters is possible by means of MS^n^ acquisition [11, 20, 21]. As mentioned above, the abiotic transformation may lead to the formation of more toxic TPs than their parent compound. Thus, the ecotoxicological impact of the analytes and their TPs was assessed by using the marine bacterium *Vibrio fischeri* and in silico by means of ECOSAR software.

## 2 Materials and Methods

### 2.1 Materials

Epoxiconazole (≥ 99%), coumarin (≥ 99.9%), and formic acid (EMSURE ACS, Reag. Ph Eur, purity 98%–100%) were purchased from Merck Sigma Aldrich (Milan, Italy), while hymecromone (≥ 99%) from European Pharmacopoeia Reference. Titanium dioxide P25 (TiO_2_) was exploited as photocatalyst. Acetonitrile (HiPerSolv CHROMANORM, purity ≥ 99.9%) was purchased from VWR Chemicals, Milan, Italy. Syringe filters (Millex LCR) in hydrophilic PTFE with a porosity of 0.45 μm from Merck Millipore were used after photocatalytic tests. All the materials were used without additional purification. All solutions were prepared in ultrapure water, produced by a Milli‐Q system (Evoqua, Pittsburg, USA). For the toxicity assessment, freeze‐dried bacteria *V. fischeri* , reconstitution solution, diluent (2% NaCl), and an adjustment solution (nontoxic 22% NaCl) were used and obtained from Azur (Milan, Italy).

### 2.2 Irradiation Procedures

To evaluate the photodegradation process, epoxiconazole aqueous solution was prepared at a concentration of 20 mg/L, while thanks to the higher solubility of coumarin and hymecromone their solutions' concentration of 40 mg/L was used. Photodegradation experiments were carried out in cylindrical Pyrex glass cells (2.3‐cm height × 4.0‐cm diameter) kept under magnetic stirring conditions. In all studied photoinduced processes, the removal kinetics of the analytes were assessed from the concentration versus time curves. The pseudo‐first order equation (Equation 1) was applied to estimate the respective rate constants of the analytes

(1) $$C_{t}=C_{0}e^{-\mathrm{kt}}$$

where *C*_*t*_ is the concentration of analyte at time t, *C*_0_ is the initial concentration, and k is the rate constant.

The evaluation of the photostability of the compounds and the formation of their TPs was performed under direct photolytic conditions or under heterogeneous photocatalysis using TiO_2_ P25 as photocatalyst.

For direct photolysis experiments, 5 mL of the solution of the analyte was subjected to light irradiation. The photocatalytic degradation of the analytes was performed in the aforementioned cells filled with 5 mL of CEC's solution and 400 ppm of TiO_2_ suspension in a ratio of 1:1, put under UV‐A light irradiation for different times. A Philips Cleo 6 × 15‐W TL‐D Actinic BL lamp with maximum emission wavelength at 365 nm was used a light source. Irradiation times were chosen from 5 min to 2 h. After irradiation, the entire content of each cell was filtered through a 0.45‐mm filter and analyzed by HPLC‐HRMS. The degraded samples of epoxiconazole were freeze‐dried before HPLC‐HRMS analysis by using a CoolSafe 55/100 (Montepaone SRL, Turin, Italy). The freeze‐dried product was dissolved in a mixture of 70:30 (v/v) of acetonitrile and 0.1% formic acid in water.

### 2.3 HPLC‐HRMS

The CECs removal and the identification of their TPs were followed by high performance liquid chromatography (HPLC) coupled to high resolution mass spectrometry (HRMS). A Thermo Scientific (Milan, Italy) UltiMate 3000 Basic Automated System chromatograph coupled with a Orbitrap Fusion Tribrid high resolution mass analyser via a H‐ESI (Electrospray Ionisation) type ion source was used. The chromatographic separation was achieved by using a C18 Phenomenex (Bologna, Italy) Luna column (15‐cm length × 2‐mm diameter), consisting of particles with a diameter of 3 μm and pore sizes of 100 Å and thermostated at 40°C. The method involved an injection volume of 20 μL and a flow rate of the mobile phase of 0.200 mL/min. The mobile phase was composed of 0.1% formic acid in water (eluent A) and acetonitrile (eluent B). Gradient separation ramp started with 5% B and increased up to 100% B in 30 min.

The mobile phase was transferred to ESI ion source by using nitrogen both as sheath and auxiliary gas. Source parameters were set as follows: sheath gas 35 arb (arbitrary unit), auxiliary gas 21 arb, capillary voltage 4.0 kV, and capillary temperature 275°C. Full mass spectra were acquired in positive ion mode in the *m/z* range between 100 and 700 for epoxiconazole and between 50 and 500 for hymecromone and coumarin, with a resolution of 50 k. MS^n^ spectra were acquired in the range between ion trap cut‐off and precursor ion *m/z* values. Precursor ions were focused on a window of 5 *m/z* for epoxiconazole, which is a chlorinated species, and 3 *m/z* for hymecromone and coumarin.

For structural elucidation of TPs, we used the Xcalibur 4.0 (version 4.0.27.13, Thermo Scientific) software data based on mass accuracy (< 5 ppm) and RDB (ring double bond) index. Accurate *m/z* values and isotopic abundance in full mass scan chromatogram were used to identify elemental composition, whereas MS^n^ fragmentation was used to hypothesize structural modifications. We followed the Shymanski rules to assign the identification level for the recognized TPs [22]. Therefore, it was possible to characterize several TPs through MS^n^ acquisition and recognize the probable abiotic transformation pathways, which could occur in the euphotic zone of the surface water. The annotation and the characterization of TPs were carried out on the solutions spiked with TiO_2_ and irradiated for 15 min, when the maximum of their production occurred. The TPs were characterized by assessing their evolution kinetics, chromatography behaviour, accurate mass, and MS^n^ spectra. For each compound, the information derived from MS^2^ and MS^3^ was compared with the fragmentation pathways of the parent substances to assign the tentative structures for most of the TPs.

### 2.4 Toxicity Assessment

The acute toxicity of epoxiconazole, hymecromone, and coumarin and their TPs was assessed according to the *V. fischeri* bioluminescence assay [2]. A Microtox Model 500 toxicity analyser (Milan, Italy) was used to monitor changes in the natural emission of the luminescent bacteria by means of the ECOSAR model v2.0. Samples were evaluated in a medium containing 2% NaCl and the degree of bioluminescence inhibition of the marine bacterium *V. fischeri* was recorded after 5, 15, and 30 min of incubation at 15°C [23].

ECOSAR (Ecological Structure Activity Relationships) software v2.2 was used for in silico toxicity prediction of the three analytes and their TPs for three organisms (fish, daphnid, and green algae). The predicted acute and chronic toxicities of TPs were computed by drawing the TP chemical structure or inserting Simplified Molecular Input Line Entry System (SMILES) strings. ECOSAR provides values of toxicity according to four levels: a range from 0.0 to 1.0 indicates high toxicity, from 1 to 10 denotes toxicity, from 10 to 100 suggests harmful impacts, and values over 100 are classified as nontoxic [24].

## 3 Results and Discussion

### 3.1 CECs Photoinduced Degradation

**FIGURE 2:**
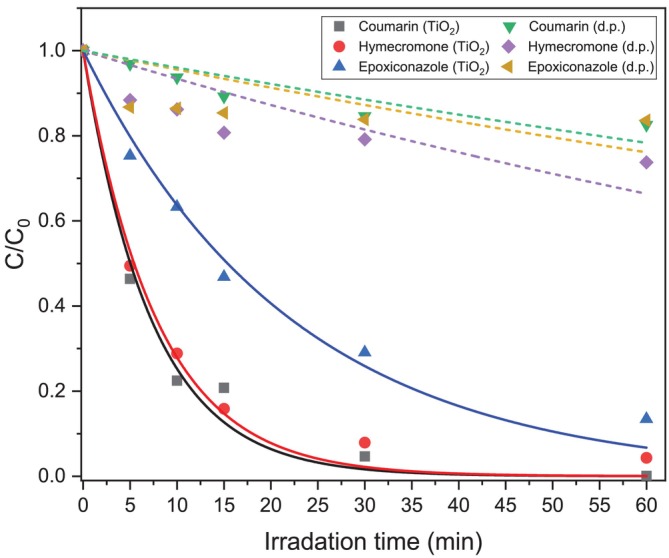
Kinetics of direct photolysis (d.p., dash line) and photocatalysis (TiO_2_, solid line) of epoxiconazole, coumarin, and hymecromone under UV‐A light.

The photoinduced removal of the investigated CECs (epoxiconazole, coumarin, and hymecromone) was assessed, and their abatement profiles are reported in Figure 2, while the calculated rate constants (k) are collected in Table 1. Direct photolysis promoted a tiny degradation of the three CECs within 60 min of irradiation and around 20% of analytes disappeared. After the addition of TiO_2_, epoxiconazole exhibited a half‐life time (t_½_) of 15 min, while for coumarin species was 5 min.

**TABLE 1:** Degradation kinetics parameters of CECs under different photoinduced degradation conditions.

CECs	Photoinduced process	t_½_ (min)	k (min^−1^)
Epoxiconazole	d.p.	165	0.004
	TiO_2_	15	0.045
Coumarin	d.p.	178	0.007
	TiO_2_	5	0.134
Hymecromone	d.p.	110	0.005
	TiO_2_	5	0.128

### 3.2 TPs Characterization by HRMS

To identify and characterize the chemical structures of the TPs formed from the three CECs after the photoinduced process, the MS^n^ fragmentation pathway for the three parent compounds was comprehensively investigated. The TPs structural characterization was accomplished by comparing the parent compound MS^n^ spectra with TPs fragmentation pathways.

**FIGURE 3:**
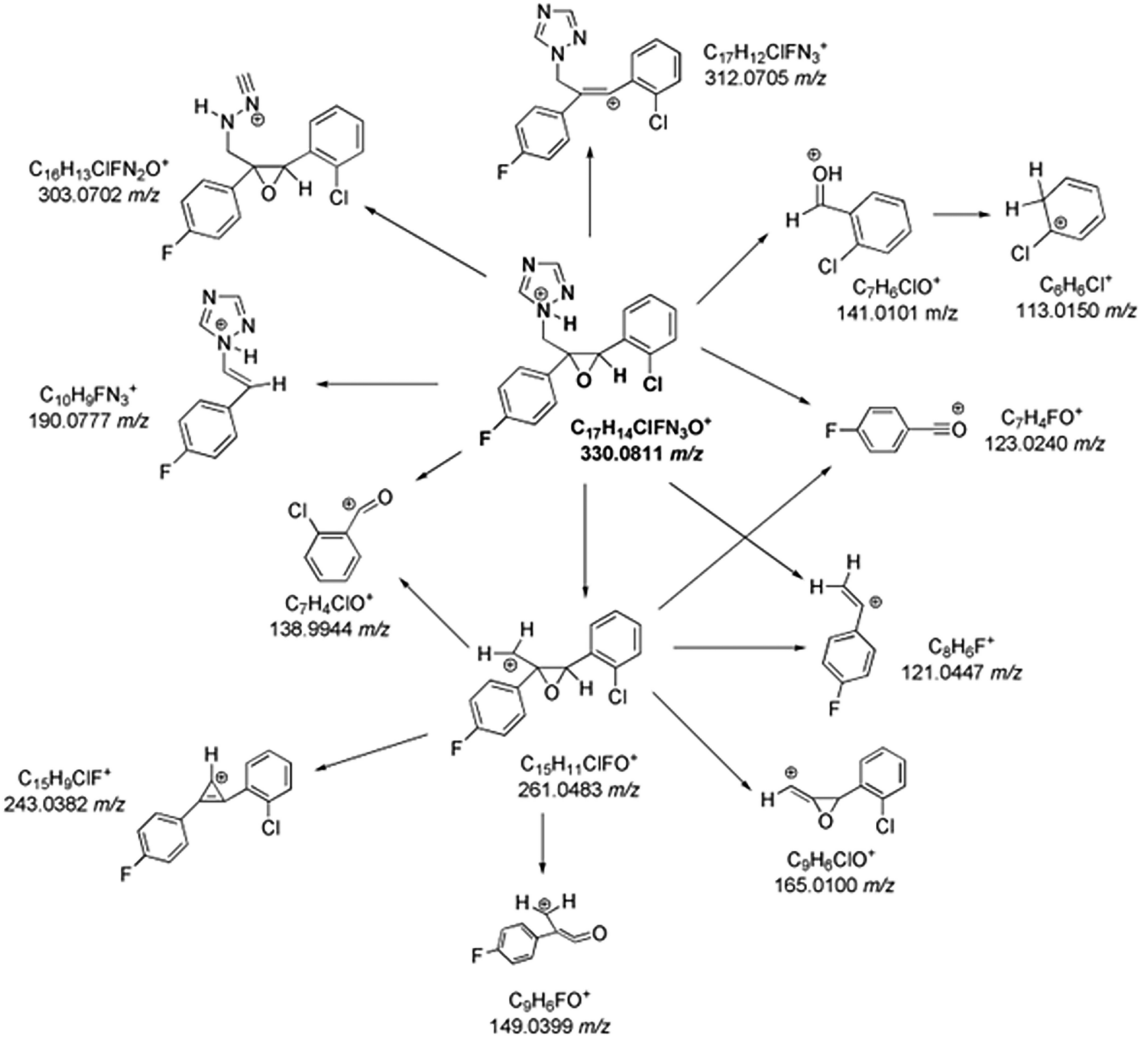
MS^n^ fragmentation pathways for epoxiconazole.

Considering epoxiconazole, the MS^2^ spectrum evidenced several product ions (Table S1) (Figure S1), and the proposed fragmentation pathways are shown in Figure 3. In particular, two structurally‐diagnostic product ions of epoxiconazole were identified: the base peak at 121.0446 *m/z*, formed through the loss of a methyl‐1,2,4‐triazole and chlorobenzene, and the acylium ion bound to the chlorophenyl moiety at 138.9944 *m/z*. The signal at 312.0705 *m/z* was recognized as a result of the loss of an H_2_O molecule from the epoxiconazole protonated molecular ion. The epoxy ring could also be involved in several fragmentations. For instance, the abundant C_7_H_6_ClO^+^ ion (141.0101 *m/z*) was produced and could generate the product ion at 113.0150 *m/z* due to the loss of CO in MS^3^. In addition, the epoxy ring opening led to the formation of C_10_H_9_N_3_F^+^ at 190.0777 *m/z* due to the neutral loss of chlorobenzaldehyde with the subsequent formation of a double bond. Moreover, the 1,2,4‐triazole ring of epoxiconazole broke with the loss of a HCN molecule, as the peak at 303.0702 *m/z* confirmed with a low abundance. The main fragmentation pathway was the elimination of the 1,2,4‐triazole to produce the C_15_H_11_OClF^+^ ion at 261.0483 *m/z*. However, due to its nature of secondary carbocation, C_15_H_11_OClF^+^ ion shows a low abundance (1%, Table S1), and the MS^2^ characteristic peaks at 138.9944 *m/z*, 123.0240 *m/z*, and 121.0447 *m/z* were identified from its MS^3^ spectra assessment.

**FIGURE 4:**
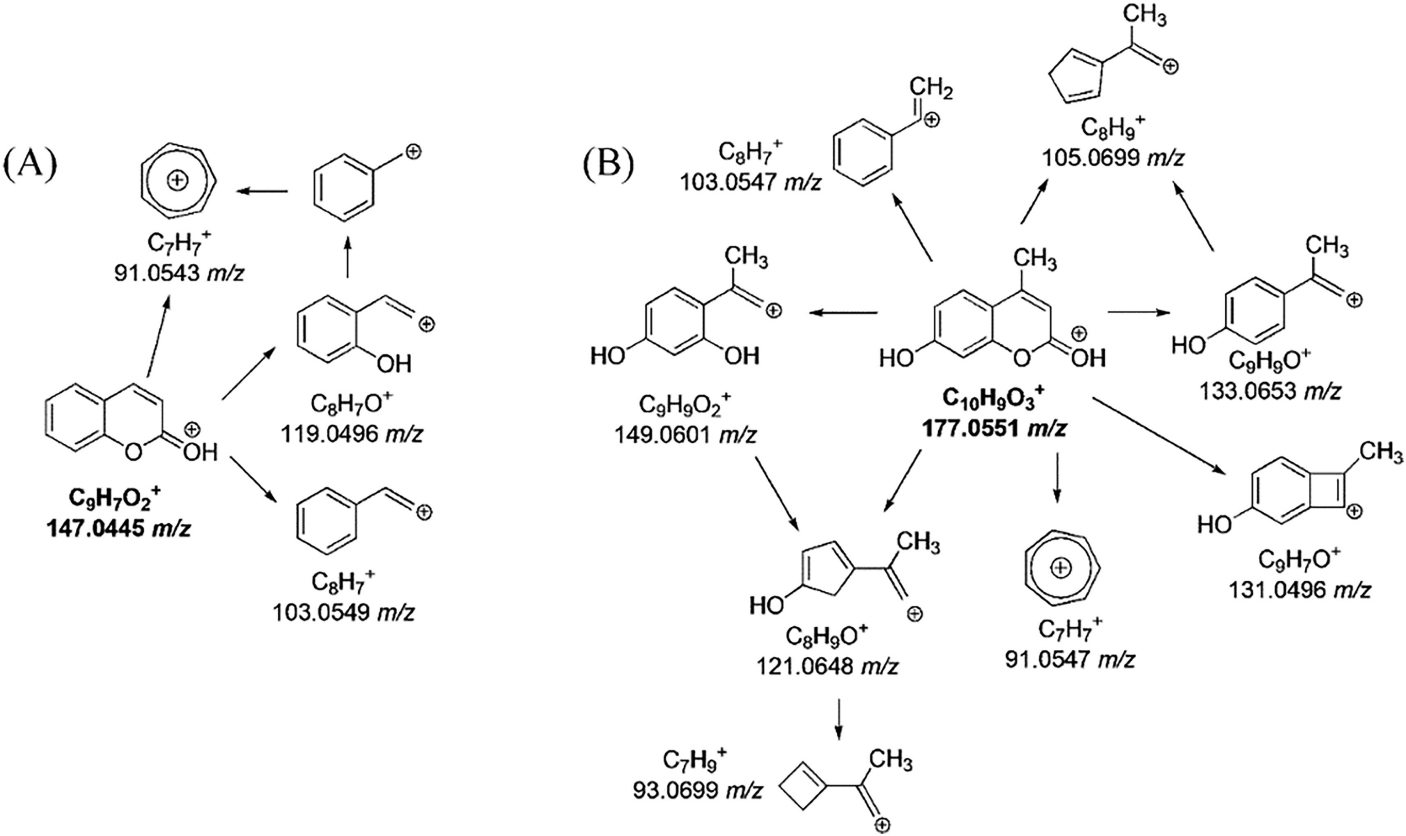
MS^n^ fragmentation pathways for coumarin (A) and hymecromone (B).

The MS spectra of coumarin and hymecromone showed the protonated ions at 147.0442 *m/z* and 177.0545 *m/z*, respectively (Figure S2). As shown in Table S2, and illustrated in Figure 3, both coumarin and hymecromone mostly underwent the neutral losses of CO and CO_2_. Indeed, the loss of CO_2_ from coumarin led to the base peak at 103.0549 *m/z*, while the tropylium ion (C_7_H_7_^+^) was formed from the loss of two molecules of CO (Figure 4A). As a coumarin derivative, the fragmentation pathway of hymecromone exhibited some similarities with the coumarin one, as shown in the Figure 4B. The MS^2^ spectrum of hymecromone displayed two characteristic signals of coumarin, 103.0547 *m/z* and 91.0547 *m/z*, despite their low abundance. The typical losses of CO and CO_2_ from hymecromone produced the peaks at 149.0601 *m/z* and 133.0653 *m/z*, respectively. As MS^3^ study confirmed (Table S2), hymecromone fragments underwent subsequent losses of CO. Finally, the product ion at 131.0496 *m/z* was formed by formic acid loss.

### 3.3 Formation of TPs

The identification of unknown TPs was performed through the comparison of HPLC/ESI‐HRMS chromatograms before and after 15 min of photocatalytic degradation. Most of the photoinduced TPs of the investigated CECs were detected in the form of structural isomers, due to the non‐selectivity of ^•^OH radicals [18], involved in the heterogeneous photocatalysis process. The accurate mass for all TPs, and thus their corresponding empirical formulae, was established and through an in‐depth analysis of MS^2^ and MS^3^ spectra. Therefore, a probable structure (Level 2 of identification confidence) was assigned to most of TPs, while tentative structural candidates were ascribed especially to the hydroxylated TPs due to insufficient information for one exact structure (Level 3 of identification confidence) [22].

#### 3.3.1 Epoxiconazole TPs

Twenty‐seven TPs were detected during the photoinduced transformation of epoxiconazole. Figure S3 depicts their chromatographic profiles, which allowed to evaluate their temporal profiles (Figure S4). As can be seen from Figure S4, most of TPs achieved their maximum concentration in 15 min of irradiation and disappeared in 1 or 2 h, suggesting their production through different concomitant pathways. The overall TPs are presented below based on the process involved in their formation. Moreover, no new different TPs were found from direct photolysis experiments.

##### 3.3.1.1 Hydroxylation

The photocatalytic degradation of epoxiconazole showed the formation of numerous hydroxylated derivatives.

**FIGURE 5:**
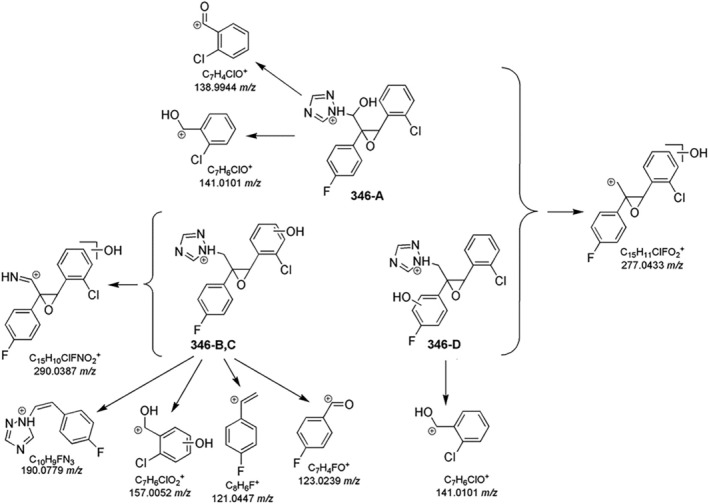
Key fragmentation pathways for epoxiconazole monohydroxy TPs 346‐A, B, C, and D.

Four monohydroxylated species originated from protonated molecules were detected at 346.0756 *m/z* (346‐A, B, C, and D, Table S3). The presence of the isotopic peak at M + 2 *m/z* (348.0722 m/z) confirmed that the chlorophenyl portion was still present after the transformation process (Figure S5). Figure 5 illustrated the main fragmentations of monohydroxy‐epoxiconazole isomers. All 346 isomers shared some fragmentations with the parent molecule, like the loss of the triazole ring (277.0433 *m/z*). Thus, the triazole ring was supposed not involved in the hydroxylation. Moreover, in the MS^2^ spectra of 346‐B, 346‐C, and 346‐D, a peak at 290.0386 *m/z* was the indication of the fragmentation involving 1,2,4‐triazole. The absence of this signal in MS^2^ spectrum of 346‐A suggested the effective monohydroxylation on epoxiconazole C1 carbon (Figure 1A). The characteristic peak at 141.0101 *m/z* present in 346‐A and 346‐D MS^2^ spectra indicated that the chlorophenol portion was not involved in hydroxylation, as confirmed by the acylium ion at 138.9944 *m/z*. MS^2^ investigation on 346‐B and 346‐C, showing 123.0239 *m/z* and 121.0447 *m/z*, indicated that fluorophenyl ring was not hydroxylated. Thus, the chlorophenyl ring of 346‐B and 346‐C products was subjected to hydroxylation, as their MS^2^ peaks at 157.0052 *m/z*, corresponded to hydroxylated chlorophenylmethyl ion, and at 190.0779 *m/z* from the complementary neutral loss of hydroxychlorophenylmethanol.

**FIGURE 6:**
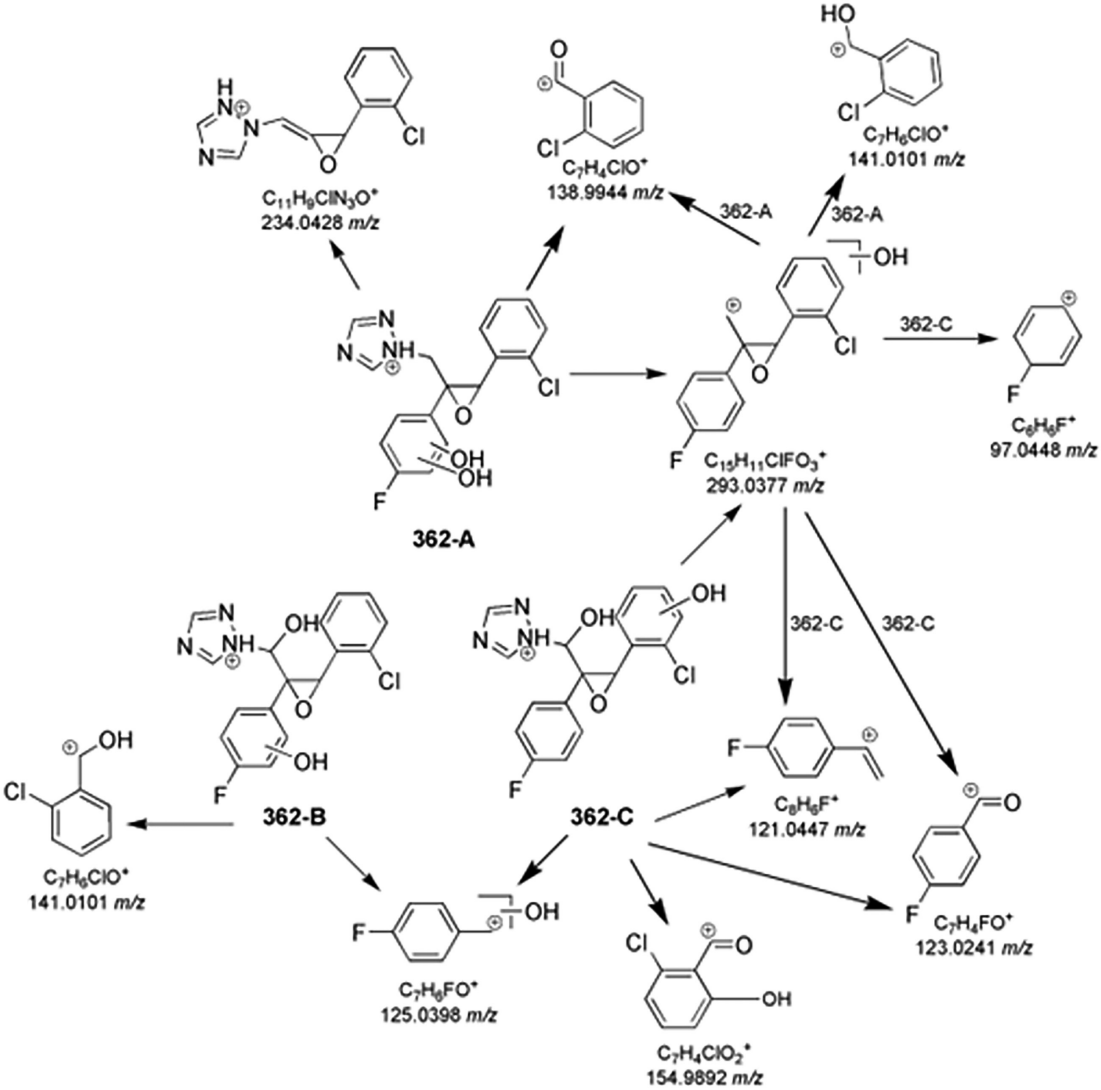
Key fragmentation pathways for epoxiconazole TPs at 362.0702 *m/z*.

The photoinduced transformation of epoxiconazole further occurred via dihydroxylation, as indicated by the three isomers at 362.0702 *m/z* (362‐A, B, and C, Table S3). Some MS^n^ ions were common to those of the parent epoxiconazole fragmentation. In 362‐A MS^2^ spectrum, the base peak at 138.9945 *m/z* and the peak at 234.0428 *m/z* implied the absence of OH groups on the chlorophenyl moiety, as well as in 362‐B due to the peak at 141.0101 *m/z*. Moreover, MS^3^ study on 362‐A fragment ion 293.0377 *m/z* confirmed the dihydroxylation on fluorophenyl moiety (Figure 6). For 362‐B, the low abundant but diagnostic fragment at 250.0376 *m/z* was produced by the neutral loss of hydroxyfluorophenol, indicating the monohydroxylation of fluorophenyl ring. The product ion at 125.0398 *m/z* implied that chlorophenyl ring was not hydroxylated. Therefore, the second OH group should be on C1 (Figure 6). On the contrary, for 362‐C, the fluorophenyl portion was not involved in hydroxylation, as confirmed by the presence of 123.0241 *m/z*. The distinctive peak of 362‐C at 154.9892 *m/z* suggested that the chlorinated aromatic ring was involved in the hydroxylation reaction (Figure 6).

**FIGURE 7:**
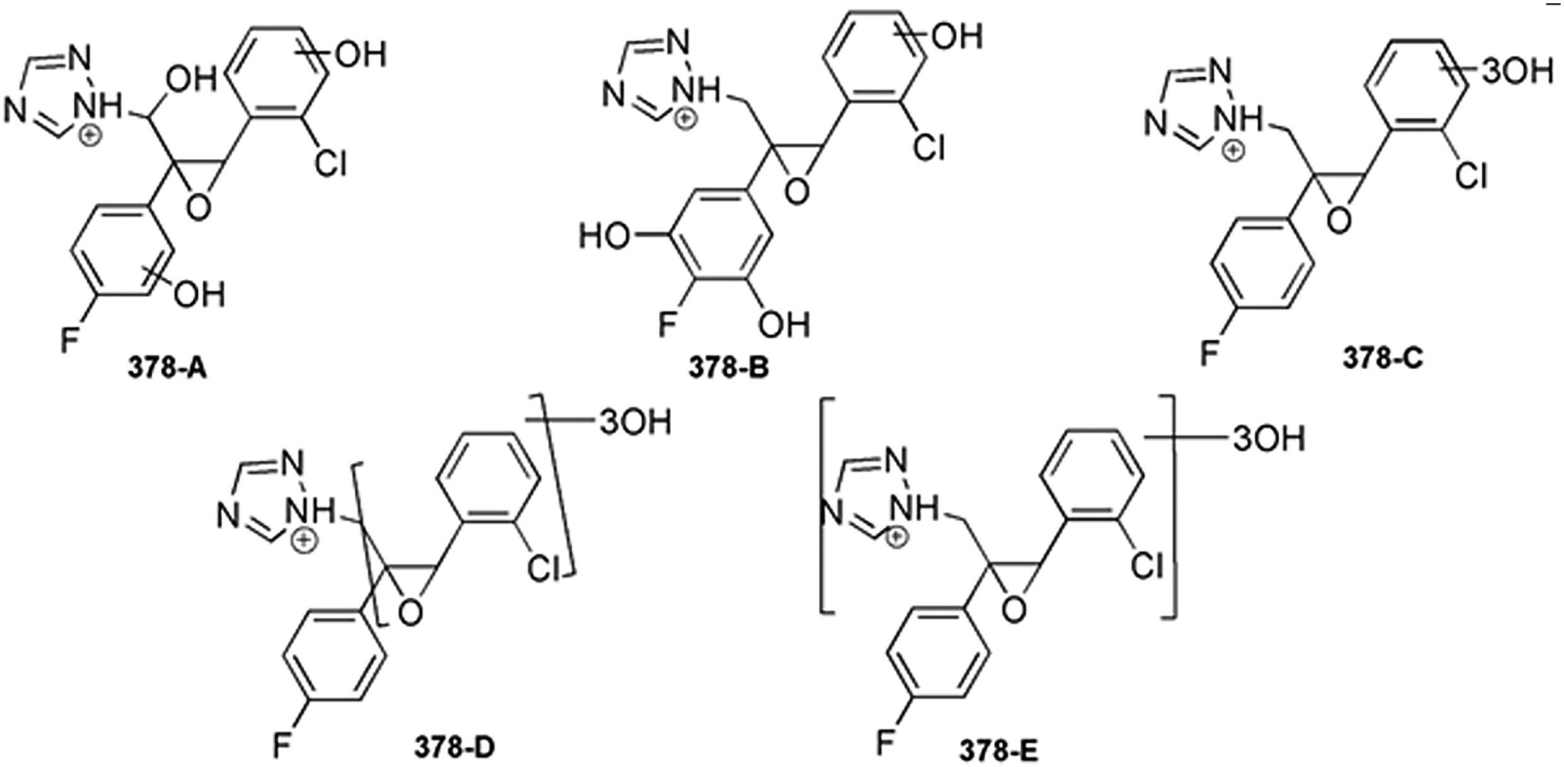
Proposed structures for 378.0652 *m/z*.

Besides, five isomers at 378.0652 *m/z* were detected as a result of the trihydroxylation (Table S3). The MS^2^ peaks formed by the fragmentation of 378‐A at 154.9892 *m/z* and 157.0059 *m/z* suggested a monohydroxylation of the chlorophenyl ring, as well as of the fluorophenyl one due to 165.0344 *m/z*. Thus, 378‐A was the product of a trihydroxylation reaction on C1 and both aromatic systems (Figure 7). Some 378‐B product ions were common with 378‐A MS^2^ fragmentation, suggesting the introduction of two OH groups on both aromatic rings. Indeed, the diagnostic ion at 141.0344 *m/z* indicated the dihydroxylation of fluorophenyl moiety and the monohydroxylation of the chlorophenyl. As far as MS^2^ of 378‐C and 378‐D is concerned, the fragment ions at 123.0239 *m/z* and 121.0447 *m/z* implied that no OH groups are bonded to fluorophenyl ring. The characteristic signal at 160.9997 *m/z* of 378‐C suggested a trihydroxylation on the chlorinated ring of epoxiconazole. The isomers 378‐D and 378‐E showed similar MS^2^ fragmentation. However, the loss of triazole ring from 378‐D (product ion at 309.0320 *m/z*) excluded 1,2,4‐triazole OH substitution, unlike in the case of 378‐E. In Figure 7, the chemical structures of epoxiconazole trihydroxylated TPs are proposed.

##### 3.3.1.2 Dehalogenation

**FIGURE 8:**
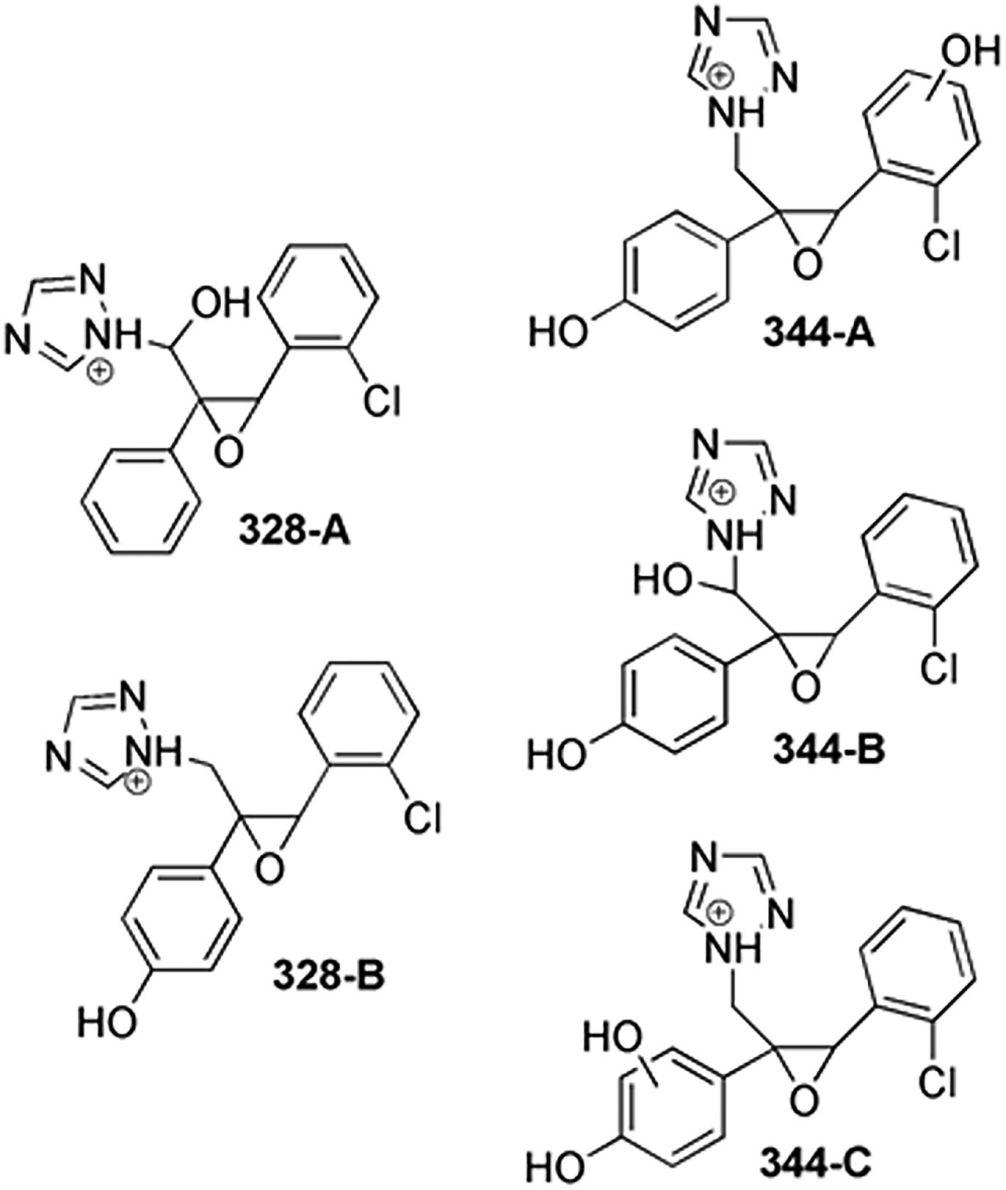
Proposed structures for dehalogenated epoxiconazole TPs.

Some TPs were formed through oxidative or reductive defluorination, as shown in Figure 8.

As can be seen in Table S4, two isomers corresponding to the peaks at 328.0850 *m/z* (328‐A, B) were detected with similar MS^2^ fragmentations. The diagnostic product ion of 328‐A at 310.0738 *m/z* was formed by the loss of an H_2_O molecule. Since H_2_O loss from an aromatic ring is unlikely, the presence of this ion suggested that 328‐A was produced through reductive dehalogenation together with a potential hydroxylation on C1 (Figure S6). The signals at 259.0515 *m/z* and 231.0569 *m/z* in MS^2^ spectrum of 328‐A confirmed its structure, according to the mechanism proposed in Figure S6. Instead, the absence of these diagnostic product ions in the spectrum of 328‐B MS^2^ was indicative of oxidative defluorination.

Three isomers were detected at 344.0806 *m/z* (344‐A, B, and C, Table S4) well‐matched with the hydroxylation of the defluorinated TPs. The fragment ion of 344‐A at 154.9892 *m/z* suggested a hydroxylation on chlorophenyl ring, further confirmed by the product ions at 216.0763 *m/z*, 188.0814 *m/z*, and 119.0490 *m/z* (Figure S7). The 344‐B product ions in common with the 328‐A MS^2^ fragmentation suggest that the dehalogenated aromatic ring was not involved in the hydroxylation (Table S4). The loss of an H_2_O molecule from 344‐B, producing the ion at 326.0677 *m/z*, suggested the C1‐hydroxylation, further confirmed by the loss of 1,2,4‐triazole. For 344‐C isomer, the hydroxylation involved the defluorinated aromatic ring, coherent with the fragment ion at 141.0100 *m/z*.

##### 3.3.1.3 Cleavage of the Molecule

Ten TPs were identified following epoxiconazole cleavage with the detachment of the chlorophenyl or fluorophenyl moieties. The MS^2^ spectrum of the TP at 206.0728 *m/z* was characterized by the absence of the typical Cl isotopic pattern. Therefore, TP 206 was formed by the loss of chlorotoluene due to epoxy ring opening with the formation of a ketone. MS^2^ product ions at 179.0612 *m/z* (loss of HCN molecule) and 137.0397 *m/z* (loss of 1,2,4‐triazole), and the MS^3^ fragmentation of 179.0612 *m/z* confirmed the chemical structure (Table S5) with the formation of this intermediate via epoxiconazole cleavage (Figure S8).

**FIGURE 9:**
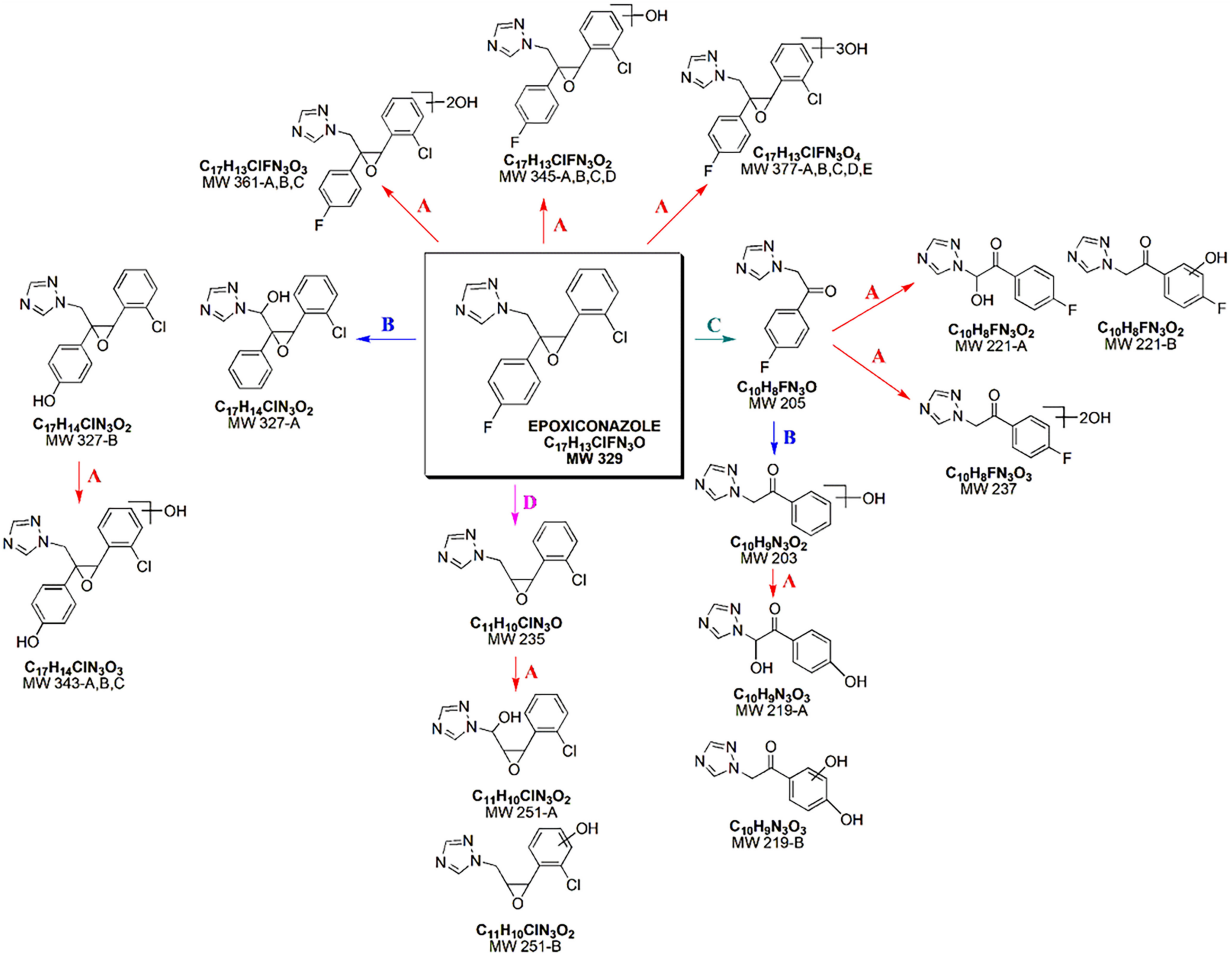
Summary of the TPs of epoxiconazole with their production pathways. (A) hydroxylation; (B) defluorination; (C) loss of chlorotoluene; (D) loss of fluorobenzene.

Moreover, TP 206 underwent subsequent transformation pathways. Indeed, four derivatives formed from TP 206 have been monitored (Figure 9). Oxidative defluorination of 206 TP produced the TP detectable at 204.0771 *m/z*. The MS^2^ product ions of TP 204 at 135.0440 *m/z* and 107.0493 *m/z* confirmed its formation pathway, involving the defluorination of TP 206 followed by the hydroxylation (Table S5).

Two isomers at 220.0718 *m/z* formed via hydroxylation of TP 204 were identified. As shown in Table S5, the MS^2^ peak at 123.0439 *m/z* and 95.0491 *m/z* for 220‐A suggested that the aromatic ring was not involved in hydroxylation. The loss of the triazole ring producing the fragment ion at 151.0387 *m/z* proved that the OH group is bonded to C1 of TP 220‐A. In contrast, 220‐B presents two OH groups on the aromatic ring, one resulting from oxidative dehalogenation and the other one from hydroxylation, as suggested by its diagnostic product ion at 111.0440 *m/z*.

Finally, TP 206 underwent monohydroxylation, with the formation of two isobaric species at 222.0672 *m/z* and dihydroxylation (238.0627 *m/z*, see Table S5). 222‐B was produced by monohydroxylation on the aromatic ring as inferred by the base peak at 139.0188 *m/z* and its product ion at 111.0241 *m/z*. On the contrary, the OH group in 222‐A was bonded to C1, as revealed by its product ions at 153.0347 *m/z* (loss of 1,2,4‐triazole) and 123.0241 *m/z*. TP 206 was also dihydroxylated, as shown by the TP at 238.0627 *m/z*. The effective dihydroxylation of TP 206 was confirmed in the mass spectrum of TP 238 by the presence of the fragment ion at 169.0293 *m/z* and the loss of C_3_H_3_ON_3_, which gave a product ion at 141.0345 *m/z*. No isomers of TP 238 were identified, suggesting that coelution may have occurred.

Epoxiconazole degradation then led to the cleavage of the fluorinated moiety, as proved by the formation of the TP at 236.0583 *m/z*, still presenting the typical isotopic pattern of chlorine (Figure S9 and Table S5). It shares several fragmentations with the parent compound, such as the triazole loss (69.0323 *m/z*) and the product ion at 138.9944 *m/z*.

Lastly, two isomers at 252.0540 *m/z* formed through monohydroxylation of TP 236 were detected. The aromatic ring of 252‐A was not involved in the hydroxylation, as confirmed by the fragment ions at 138.9944 *m/z*, 137.0151 *m/z*, and 113.0153 *m/z*. In the case of 252‐A, the base peak at 183.0204 *m/z* due to the loss of 1,2,4‐triazole revealed the OH group C1‐position. In contrast, the diagnostic peaks of 252‐B MS spectrum at 154.9888 *m/z* and 110.0348 *m/z* proved the monohydroxylation had occurred on the chlorophenyl moiety.

##### 3.3.1.4 Transformation Pathways

The neutral forms of the TPs of epoxiconazole were shown in Figure 9 and labelled based on their molecular weight. Based on the proposed structures and their evolution/degradation profiles, several oxidative and reductive concomitant pathways occurred:

- (Poly)hydroxylation (path A)
- Defluorination (path B) with following formation of secondary TPs via hydroxylation
- Loss of chlorotoluene (path C) with following formation of TPs via hydroxylation and dehalogenation
- Loss of fluorobenzene (path D) with subsequent hydroxylation.

#### 3.3.2 Coumarin and Hymecromone TPs

The photoinduced degradation of coumarin proceeded through the formation of six TPs, while in the case of hymecromone, eight TPs were detected. In the photolytic degradation of hymecromone, a new TP was identified at 209.0441 *m/z*. This hymecromone derivative was not detected after the photocatalytic experiment probably due to its rapid transformation. Figures S10 and S11 show the chromatographic profiles of TPs of coumarin and hymecromone, while their temporal evolution profiles are reported in Figures S12 and S13. Most of the coumarin and hymecromone TPs were easily formed after 5 min of irradiation and they disappeared within 2 h. The empirical formulas of the identified TPs of coumarin and hymecromone are collected in Tables S6 and S7, respectively. A tentative structure (Level 3 of identification confidence) was assigned to (poly)hydroxylated TPs and their derivatives, while the chemical structures of the other TPs, coumarin and hymecromone were assessed with a Level 2 of identification confidence [22].

##### 3.3.2.1 Hydroxylation

Coumarin and hymecromone photocatalytic degradation produced species originating [MH]^+^ ions, which differed from the parent molecule by 15.9948 and 31.9898 u, well‐matched with mono‐ and dihydroxylation.

The photocatalyzed process mainly formed the monohydroxylated derivatives of both coumarin and hymecromone. Three isomers of monohydroxylated coumarin (163‐A, B, and C, Table S6) were detected at 163.0386 *m/z*, while two isomers at 193.0482 *m/z* resulted from the monohydroxylation of hymecromone (193‐A and B, Table S7).

**FIGURE 10:**
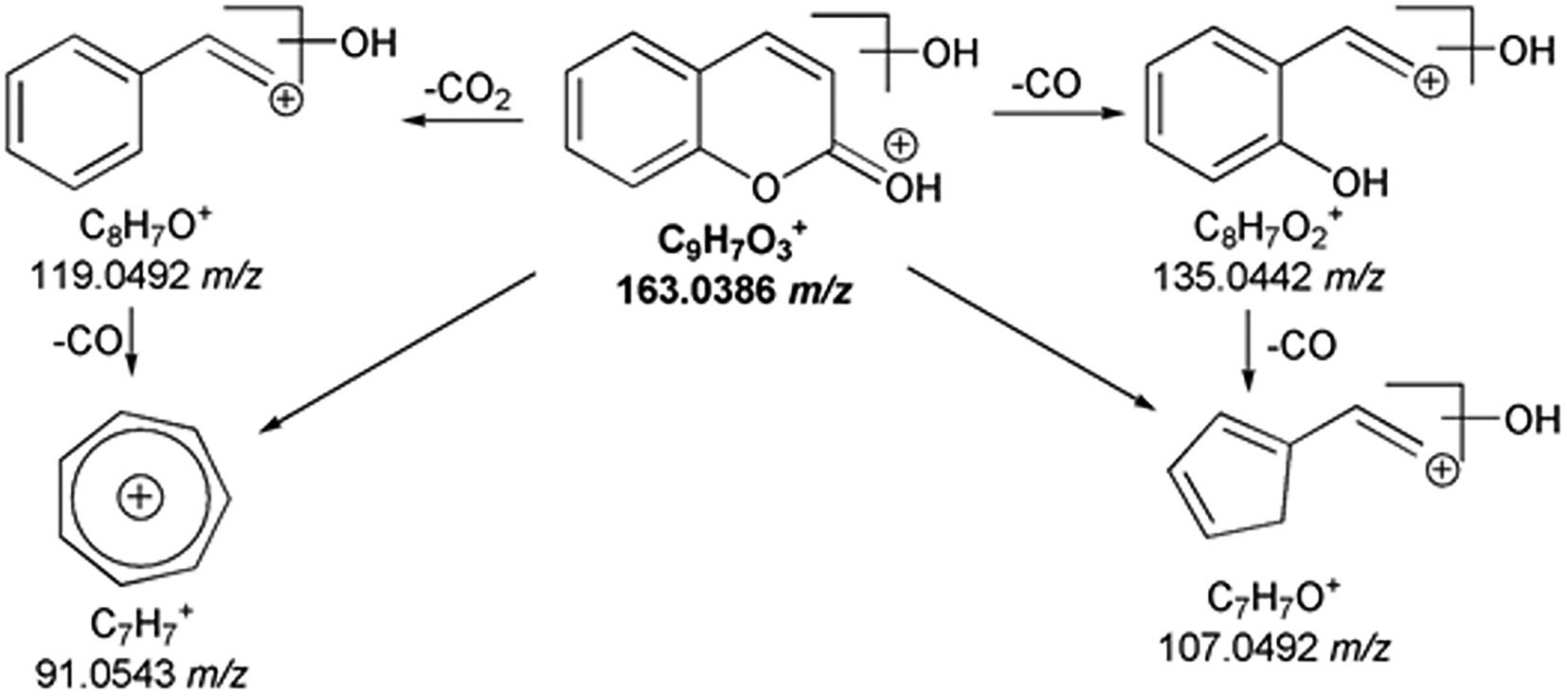
MS^n^ fragmentation pathways of mono‐hydroxylated products of coumarin.

The overall fragmentation scheme of monohydroxylated TPs of coumarin is reported in Figure 10. The three monohydroxylated TPs (163 A, B, and C) displayed the same fragmentation pathways, so preventing the possibility to univocally locate the OH group. The two MS^2^ ions at 119.0492 *m/z* and 91.0543 *m/z* were formed by the loss of a molecule of CO_2_ and by the joint losses of CO and CO_2_, respectively. The loss of a molecule of CO from the isomers of TP 163 produced the ion at 135.0442 *m/z*, which by MS^3^ fragmentation underwent a second loss of CO, providing the signal at 107.0492 *m/z* (Table S6).

**FIGURE 11:**
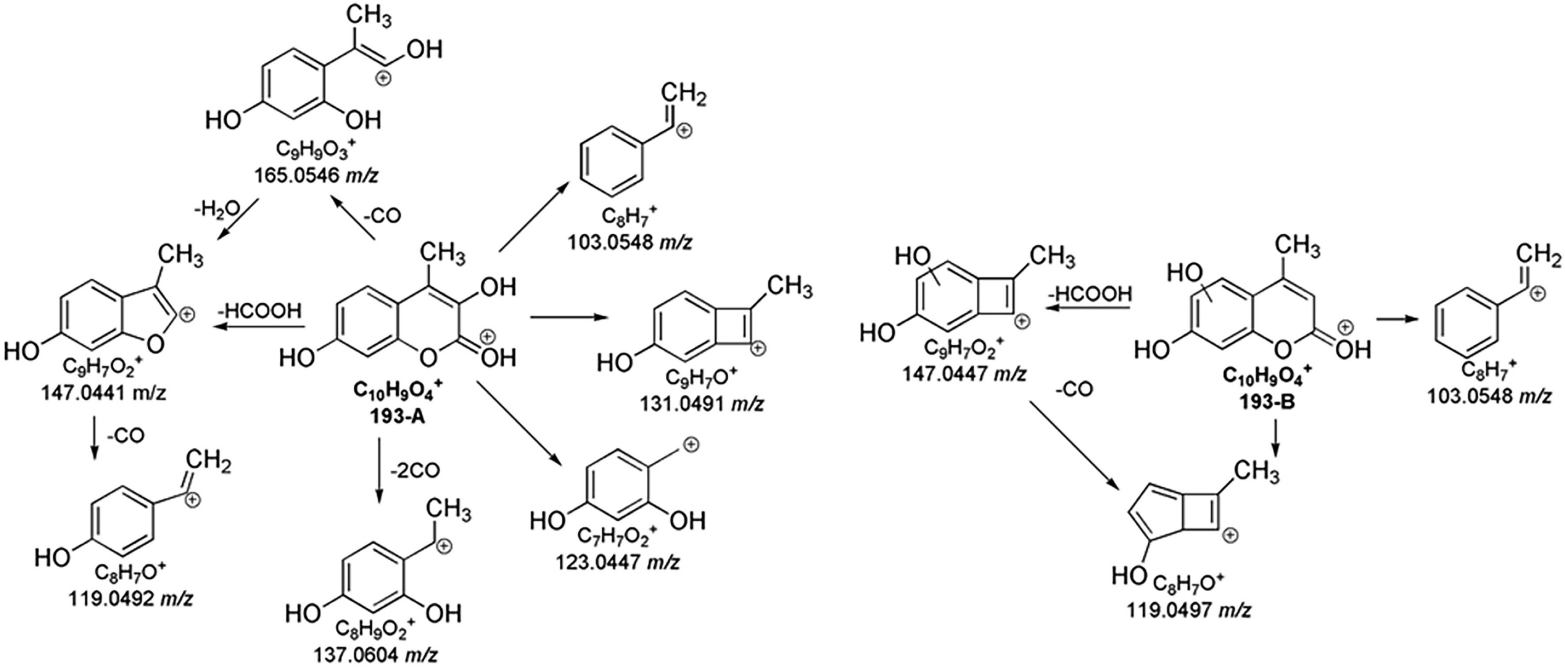
MS^n^ fragmentation pathways for isomers A and B of monohydroxylated products of hymecromone.

The MS^n^ fragmentations of the two isomers of monohydroxylated product of hymecromone (193‐A and B) are provided in Table S7 and illustrated in Figure 11. Both isomers generating ions at 193 *m/z* fragmented into the product ion at 103.0548 *m/z*, which was identified in MS^2^ spectrum of parent hymecromone as well (Table S2). The formation of the product ions of TP 193‐A at 165.0546 *m/z*, 137.0604 *m/z*, and 123.0441 *m/z* allowed to place the hydroxyl group on the carbon adjacent to the carbonyl group, named C3 (Figure 11). The ion at 165.0546 *m/z* in the MS^2^ spectrum of 193‐A due to the CO loss was a carbocation stabilized by resonance thanks to the sharing of the oxygen electron pairs. Additionally, MS^3^ fragmentation of 165.0546 *m/z* formed the ion at 147.0441 *m/z*, which was also present in the MS^2^ spectrum of 193‐A due to the direct loss of a formic acid molecule (Figure S14). As the mechanism reported in Figure S15 shows, the peak at 123.0441 *m/z* was produced by the loss of propiolic acid from 193‐A, while the loss of two molecules of CO with subsequent lactone opening produced the ion at 137.0604 *m/z*. Finally, the fragmentation of 193‐A only produced the product ion at 131.0498 *m/z* (Figure S15), a signal already observed in the MS^2^ of hymecromone, confirming the monohydroxylation on C3. The absence of these diagnostic MS^n^ ions in MS spectra of TP 193‐B is well‐matched with the monohydroxylation on the aromatic ring (Figure 11). 193‐B lost a molecule of formic acid (147.0441 *m/z*) followed by the loss of a CO molecule in MS^3^ experiment providing the product ion at 119.0492 *m/z* confirming the OH location (Figure S16).

**FIGURE 12:**
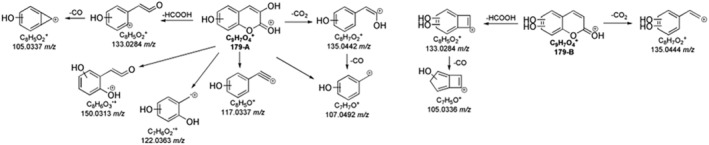
MS^n^ fragmentation pathways for isomers A and B of dihydroxylated products of coumarin.

Two species, 179‐A and B, were identified and attributed to dihydroxylated intermediates of coumarin (179.0336 *m/z* and 179.0333 *m/z*, respectively) (Table S6). Their MS^2^ spectra presented the product ion at 135.0441 *m/z* produced by the loss of CO_2_. Moreover, the mass difference of 15.9948 u, compared to the fragment ion at 119.0496 *m/z* of coumarin, confirmed the position of the hydroxyl group (Figure 12).

In the MS^2^ spectrum of 179‐A, several diagnostic product ions were identified. Firstly, the radical ion at 150.0313 *m/z* was formed by the loss of a formyl radical (Figure S17). Another radical loss led to the formation of the peak at 122.00363 *m/z*, whose molecular formula agreed with the presence of two oxygen atoms, one deriving from lactone ring opening and the other one due to hydroxylation (Figure S17). These ions suggested that for 179‐A, a hydroxylation occurred on the aromatic ring, and the second one on the C3‐C4 double bond. Furthermore, the simultaneous loss of CO_2_ and H_2_O with the subsequent formation of the peak at 117.0337 *m/z*, observed in 179‐A MS^2^ spectrum only, confirmed the hydroxylation of the pyran ring (Figure S18). Finally, the CO_2_ and CO losses from 179‐A originated in a hydroxy‐tropylium ion at 107.0491 *m/z* (Figure S18) in agreement with the proposed structure. The peak at 135.0441 *m/z* was detected in MS^2^ spectra of both 179‐A and 179‐B TPs, but with significantly different intensity (Table S6). Indeed, it was the base peak of 179‐A due to its high resonance stability, while the lower abundance of the same signal in 179‐B MS^2^ study was the consequence of a vinyl carbocation structure. The absence of the diagnostic product ions, like hydroxy‐tropylium ion in 179‐B MS^n^ investigation, supported the hypothesis of the dihydroxylation on the coumarin aromatic ring.

The dihydroxylated product of hymecromone was detected only in the direct photolysis experiment, resulting in the formation of an ion at 209.0441 *m/z*. As shown in Table S7, the peak at 165.0544 *m/z* matched the hydroxylated fragment ion of hymecromone at 133.0653 *m/z*. The diagnostic signal at 191.0336 *m/z* was produced by H_2_O loss, suggesting that one of the two hydroxyl groups was not bonded to the aromatic ring. Lastly, the loss of C_2_H_4_O_2_ confirmed the monohydroxylation on the hymecromone aromatic ring. However, the acquired information is not specific enough to determine the position of the second hydroxylation.

##### 3.3.2.2 Ring‐Opening Reaction/Oxidation

The photocatalytic degradation of both coumarin and hymecromone was found to involve oxidation processes occurring simultaneously with the opening of the lactone ring.

A coumarin TP was detected at 181.0493 *m/z* (Table S6). Its MS^n^ fragments were common to monohydroxycoumarin fragmentation, such as the hydroxytropylium ion. Therefore, it was hypothesized that TP 181 was formed through the monohydroxylated coumarin lactone opening. The loss of formic acid producing the peak at 135.0439 *m/z* confirmed this structural identification.

**FIGURE 13:**
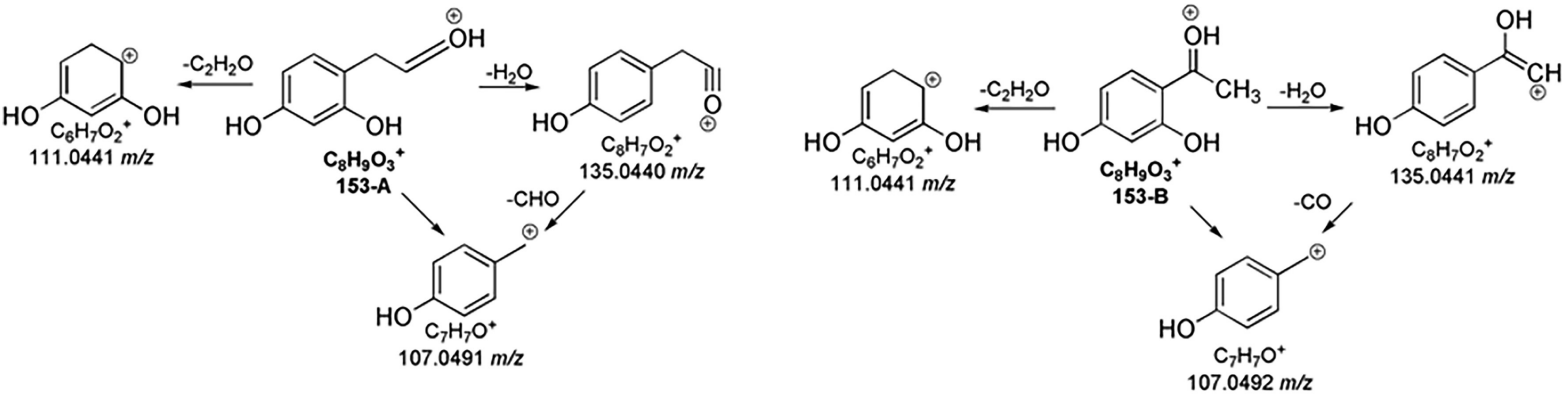
MS^n^ fragmentation pathways for hymecromone TPs 153‐A and 153‐B.

Two isobaric species were identified at 153.0541 *m/z* (Table S7). The formation of the diagnostic product ion at 111.0442 *m/z* due to ketene loss suggested the successful oxidation after the lactone ring opening of hymecromone and a simultaneous molecule cleavage. The proposed chemical structures of 153‐A and 153‐B with their fragment ions are shown in Figure 13.

##### 3.3.2.3 Demethylation/Reduction

The photoinduced transformation of hymecromone involved demethylation and a probable simultaneous reduction. Demethylation process led to the formation of the isomer 163‐C of monohydroxycoumarin, as the same retention time and coherence of MS^n^ study demonstrated (Table S7). Therefore, the TP 163‐C can be identified as 7‐monohydroxycoumarin. As can be seen in Table S7, coumarin was detected as a TP of hymecromone demethylation with the parallel reduction of its phenolic ring. Finally, demethylation together with the reduction of the C3 = C4 double bond gave rise to a TP at 165.0543 *m/z*. Its base peak at 119.0493 *m/z*, produced by formic acid loss, was diagnostic for the structure attribution (Tables S6 and S2).

##### 3.3.2.4 Transformation Pathways

**FIGURE 14:**
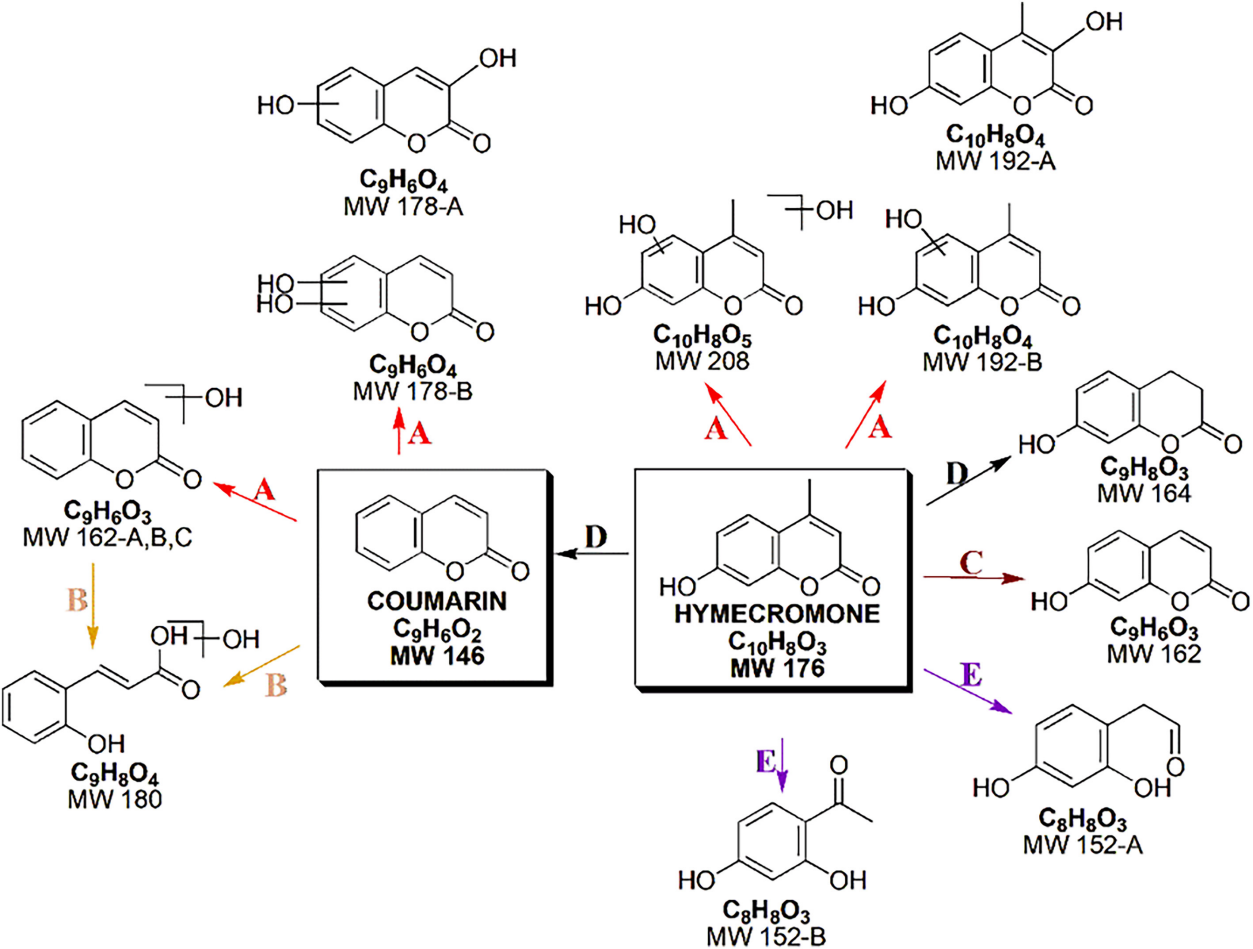
Summary of the TPs of coumarin and hymecromone with their formation pathways. (A) hydroxylation; (B) oxidative reaction following lactone ring opening; (C) demethylation; (D) demethylation with reductive reaction; (E) oxidative reaction with loss of a molecule portion.

Based on the appearance/disappearance profiles of TPs and their hypothesized structures, the photoinduced transformation processes of coumarin and hymecromone could proceed through several concomitant pathways, collected in Figure 14. For both compounds, the initial pathway involved a hydroxylation with the formation of mono‐ and dihydroxy derivatives (path A). Coumarin also underwent oxidative processes following the opening of the lactone ring (path B).

In the case of hymecromone, demethylation reactions (path C) and reductive processes (path D) could occur. A final hymecromone transformation process consisted of an oxidation reaction together with the opening of the lactone ring and the consequent loss of a portion of the molecule with the formation of two isobaric structures (path E).

### 3.4 Toxicity Evaluation

**FIGURE 15:**
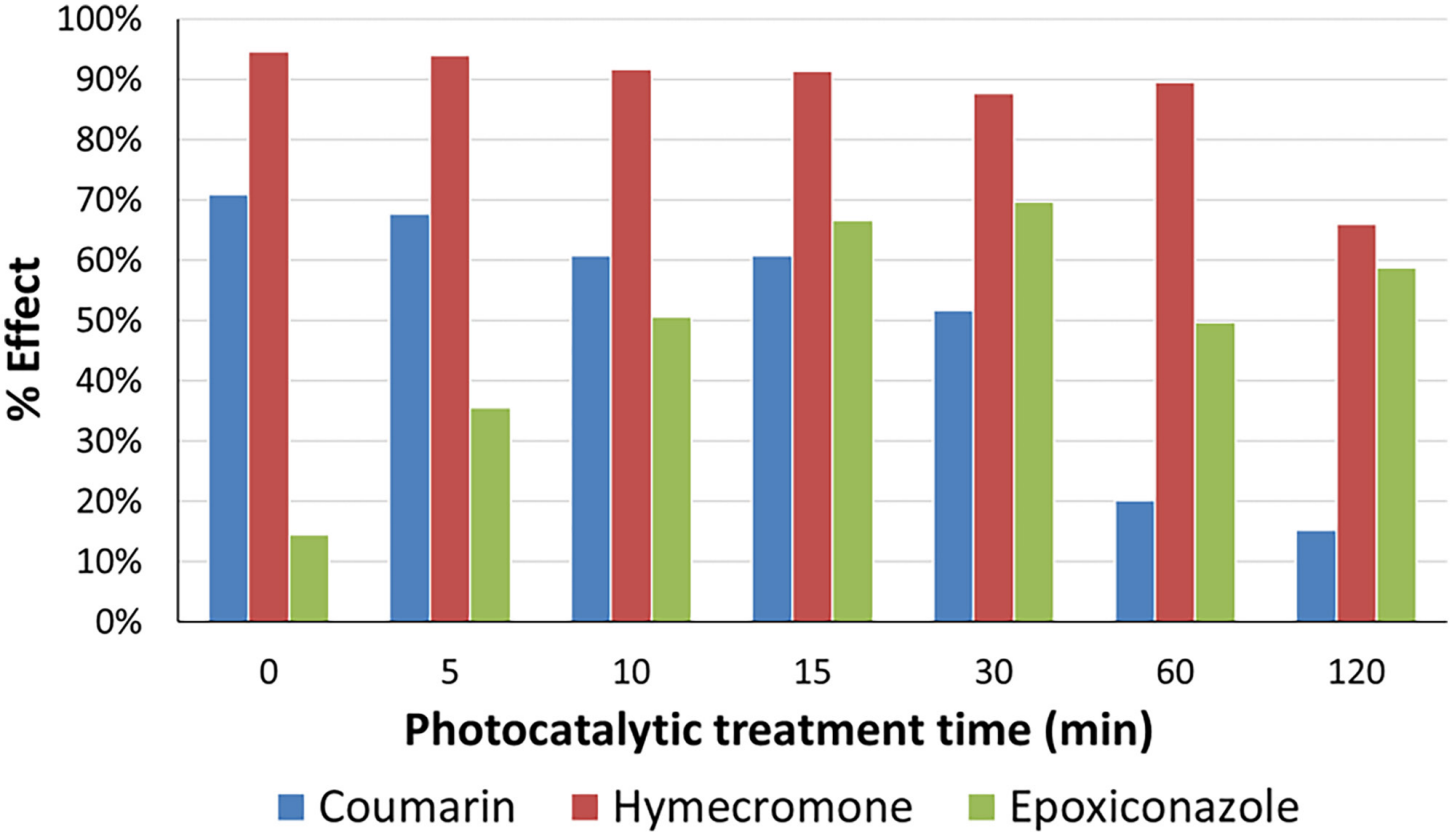
Toxicity assessed for coumarin, hymecromone, and epoxiconazole as a function of photocatalytic process time following *Vibrio fischeri* assay.

The potential acute toxicity of studied CECs and their TPs was evaluated following the *V. fischeri* assessment, monitoring the inhibition on the luminescent bacteria emission over time. Epoxiconazole is a harmless compound with an EC_50_ value greater than 20 mg/L, while coumarin and hymecromone showed EC_50_ of 10 and 0.84 mg/L, respectively. The percentage of inhibition effect on the bacteria bioluminescence by the samples resulting from the photocatalytic degradation is plotted as a function of UV‐A light irradiation time in Figure 15. The percentage of inhibition by epoxiconazole TPs reached a value of 70% after 15 min of photocatalytic treatment, together with the maximum formation of intermediates, so implying that the epoxiconazole transformation involved the production of toxic compounds. Conversely, a significant decrease in the toxicity of coumarin TPs was measured as the photocatalytic degradation proceeded, whereas hymecromone TPs still exhibited toxicity after 2 h of treatment.

In detail, as can be seen in Figure 15, the change in toxicity of epoxiconazole samples followed a similar trend to the profiles of appearance/disappearance of epoxiconazole's TPs (Figure S4). Indeed, the toxicity evaluated at time 0 corresponds to inhibition effect of epoxiconazole, while the toxicity increased as the concentrations of its TPs increased, as shown in Figure S4. After 5 min of irradiation, the increase in toxicity was caused by the formation of mono‐ and dihydroxylated products (346 and 362), the TPs formed via defluorination (328) and the loss of chlorotoluene (206) and the TPs formed by TP 206 (204, 252, 222‐A, and 238). After 10 min of photocatalytic treatment, the toxicity increased due to the contribution of the trihydroxylated products (378). Instead, after 15 min, the toxicity corresponded to the maximum formation of epoxiconazole's TPs, as mentioned above, but the contribution of isomers 220 and 344 and TPs 222‐B and 236 can be also considered. At 30 min, the toxicity was found to be high due to the maximum formation of TP 220‐A and with the contribution of TPs 206, 204, and 252‐B, whose concentrations were high as well. Although the toxicity profile decreased afterwards due to the progressive disappearance of most of the epoxiconazole TPs, after 120 min of photocatalytic treatment about 60% of inhibition effect could be partially explained by the persistent presence of TPs 206 and 204, and the probable contribution of smaller secondary TPs not detected in our experimental conditions.

Multiple species evaluation is essential to assess the toxicity of pollutants and their TPs during the photocatalytic treatment. Since the identified TPs are not commercially available, the ECOSAR program was employed as an alternative to estimate their aquatic toxicity toward fish, daphnid, and green algae. This tool allows the prediction of both acute and chronic toxicities by comparing the structural properties of substances to those of chemicals with established aquatic toxicity profiles. As a result, ECOSAR is widely applied for predicting the toxicity of compounds encountered in water treatment processes, considering the worst possible scenario [25]. The predicted values of acute and chronic toxicities of epoxiconazole, coumarin, hymecromone, and their TPs are reported in Tables 2 and 3.

**TABLE 2:** Predicted acute and chronic toxicities of epoxiconazole and its TPs.

Compound (MW)	[MH]^+^ (*m/z*)	Acute toxicity (mg/L)			Chronic toxicity ChV (mg/L)		
		Fish (LC_50_)	Daphnid (LC_50_)	Green algae (EC_50_)	Fish	Daphnid	Green algae
329 (epoxiconazole)	330	5.17	6.62	1.50	0.0073	0.404	0.745
345‐A	346	18.8	27.3	4.87	0.017	2.07	1.77
345‐B, C, D	346	9.91	6.62	2.72	0.011	0.862	1.16
361‐A	362	0.264	0.233	0.431	0.013	0.151	0.598
361‐B, C	362	12.4	8.11	3.34	0.013	1.03	1.36
377‐A	378	12.1	34.7	5.91	0.020	2.71	0.835
377‐B	378	19.3	55.7	7.71	0.026	4.68	2.81
377‐C	378	0.269	0.234	0.436	0.03	0.150	0.599
377‐D, E	378	0.294	0.269	0.490	0.015	0.182	0.697
327‐A	328	22.9	34.1	5.81	0.018	2.71	1.98
327‐B	328	12.1	7.88	3.24	0.012	0.990	1.30
343‐A	344	12.0	34.4	5.64	0.019	2.72	0.790
343‐B, C	344	15.1	9.67	3.99	0.014	1.19	1.53
205	206	392	161	19.3	0.802	17.7	4.14
221‐A	222	330	156	33.7	3.41	32.4	10.1
221‐B	222	121	35.2	14.8	0.572	3.31	6.64
237	238	0.412	0.608	0.901	0.035	0.673	1.71
203	204	152	40.6	17.1	0.695	3.68	3.79
219‐A	220	986	414	81.9	8.71	21.5	17.6
219‐B	220	99.3	292	15.4	1.46	35.0	1.64
235	236	38.8	63.0	9.10	0.023	5.83	2.50
251‐A	252	144	264	30.1	0.053	30.5	6.05
251‐B	252	75.8	40.0	16.8	0.035	3.76	3.97
			Not harmful ‐ green	Harmful ‐ yellow	Toxic ‐ orange	Very toxic ‐ red	

**TABLE 3:** Predicted acute and chronic toxicities of coumarin and hymecromone and their TPs.

Compound (MW)	[MH]^+^ (*m/z*)	Acute toxicity (mg/L)			Chronic toxicity ChV (mg/L)		
		Fish (LC_50_)	Daphnid (LC_50_)	Green algae (EC_50_)	Fish	Daphnid	Green algae
146 (coumarin)	147	34.7	84.4	46.1	2.12	10.7	13.5
162‐A, B, C	163	75.0	28.6	113	4.33	2.64	14.6
178‐A	179	0.531	1.02	1.35	0.058	1.50	1.24
178‐B	179	11.7	38.0	1.43	14.6	4.65	11.4
180	181	327	3.65 × 10^3^	75.5	206	378	9.85
176 (hymecromone)	177	38.1	92.2	49.7	2.35	11.9	14.7
192‐A	193	8.76	26.7	0.915	7.40	2.54	6.97
192‐B	193	35.4	203	8.11	4.71	24.0	0.951
208	209	13.1	42.1	1.57	15.7	5.00	12.4
164	165	162	53.7	282	8.76	4.51	30.2
162	163	75.0	28.6	113	4.33	2.64	14.6
152‐A	153	44.1	46.0	9.62	12.7	0.394	1.04
152‐B	153	16.4	157	4.74	9.75	16.6	0.588
			Not harmful ‐ green	Harmful ‐ yellow	Toxic ‐ orange	Very toxic ‐ red	

Epoxiconazole exhibited a high acute toxicity and very high chronic toxicity in all trophic levels (Table 2). As established from *V. fischeri* assay, the epoxiconazole transformation involved the production of more toxic compounds than the parent one. In particular, the higher toxicity values were assessed for the epoxiconazole TPs formed via (poly)hydroxylation. As can be seen in Table 2, all TPs, except for a few molecules, showed high chronic toxicity to fish, while no TPs were found to be chronically harmless to daphnid and green algae species. All (poly)hydroxylated epoxiconazole presented acute toxicity ranging from “harmful” to “very toxic” levels, as well as defluorinated TPs. Instead, the compounds produced via the epoxiconazole cleavage (except 237) exhibited either lower acute toxicity or harmless behaviour, especially towards fish.

Coumarin and its derivative hymecromone, were assessed as harmful (acute toxicity), while their chronic toxicity was ranged from “harmful” to “toxic” levels (Table 3). Overall, the acute toxicity of coumarin TPs was slightly higher than coumarin, but the only compound showing high acute toxicity is 178‐A towards fish, while the TP 180 was harmless towards fish and daphnids and the monohydroxylated coumarin (162‐A, B, and C) towards green algae. The same trend was assessed for the chronic toxicity. The hymecromone TPs showed an acute toxicity ranging from “harmless” to “harmful” levels towards fish and daphnids and “toxic” towards green algae (Table 3). The acute toxicity of the demethylated hymecromone (164 and 162) was assessed as innocuous for green algae but the chronic toxicity proved TPs toxic.

## 4 Conclusions

Photolysis under simulated solar radiation showed that all three species did not undergo complete degradation under UV‐A photolysis in the time window studied, whereas the heterogeneous photocatalytic process allowed significant disappearance of the analytes. By means of HPLC‐HRMS, probable or tentative structures were assigned to the TPs formed during the photocatalytic treatment of the studied compounds. The photoinduced transformation involved the hydroxylation as main photodegradative process. Epoxiconazole also underwent dehalogenation and breakage of the molecule. Coumarin compounds were affected by lactone ring opening followed by oxidation, while for hymecromone, demethylation was another preferred degradation pathway.

This study showed that the structural identification of abiotic transformation by‐products is crucial to expand the range of pollutant detection in surface waters in the future, especially in view of the risk assessment. It has to be underlined that, especially in case of epoxiconazole, its transformation proceeds through the formation of more hazardous compounds.

## Supporting information


**Figure S1.** MS^2^ spectrum of epoxiconazole with its characteristic isotopic pattern.
**Table S1.** MS^n^ of epoxiconazole.
**Figure S2.** MS^2^ spectrum of coumarin (top) and hymecromone (bottom).
**Table S2.** MS^n^ of coumarin (C_9_H_7_O_2_) and hymecromone (C_10_H_9_O_3_).
**Figure S3.** Chromatographic separation of transformation products formed from photocatalysis of epoxiconazole. NL, normalized scale.
**Figure S4.** Transformation products indicated as [MH]^+^ formed from epoxiconazole degradation as a function of the UV‐A light irradiation time in the presence of 400‐ppm TiO_2_.
**Figure S5.** Isotopic pattern of intermediate 346 of epoxiconazole.
**Table S3.** List of [MH]^+^ and fragments from MS^n^ spectra with their empirical formulas and Δppm of (poly)hydroxylated TPs of epoxiconazole.
**Table S4.** List of [MH]^+^ and fragments from MS^n^ spectra with their empirical formulas and Δppm of dehalogenated TPs of epoxiconazole.
**Figure S6.** Proposed formation mechanism of 310.0738 *m/z* and 259.0515 *m/z* with its product ion at 232.0569 *m/z* from intermediate 328‐A.
**Figure S7.** Proposed formation mechanism of 216.0763 *m/z*, 188.0614 *m/z* with its product ion at 119.0490 *m/z*, and 154.9892 *m/z* from intermediate 344‐A.
**Table S5.** List of [MH]^+^ and fragments from MS^n^ spectra with their empirical formulas and Δppm of TPs from cleavage of epoxiconazole.
**Figure S8.** Proposed formation mechanism of 179.0612 *m/z*, 151.0666 *m/z*, and 137.0397 *m/z* from intermediate 206.
**Figure S9.** Isotopic pattern of intermediate 236 of epoxiconazole.
**Figure S10.** Chromatographic separation of transformation products formed from coumarin. NL, normalized scale.
**Figure S11.** Chromatographic separation of transformation products formed from hymecromone. NL, normalized scale.
**Figure S12.** Transformation products indicated as [MH]^+^ formed from coumarin degradation as a function of the UV‐A light irradiation time in the presence of 400‐ppm TiO_2_.
**Figure S13.** Transformation products indicated as [MH]^+^ formed from hymecromone degradation as a function of the UV‐A light irradiation time in the presence of 400‐ppm TiO_2_.
**Table S6.** List of [MH]^+^ and fragments from MS^n^ spectra with their empirical formulas and Δppm of coumarin TPs.
**Table S7.** List of [MH]^+^ and fragments from MS^n^ spectra with their empirical formulas and Δppm of hymecromone TPs.
**Figure S14.** Proposed formation mechanism of 165.0546 *m/z* with its product ion at 147.0440 *m/z*, and 147.0440 *m/z* from intermediate 193‐A.
**Figure S15.** Proposed formation mechanism of 123.0441 *m/z*, 137.0604 *m/z*, and 131.0491 *m/z* from 193‐A.
**Figure S16.** Proposed formation mechanism of 147.0447 *m/z* with its product ion at 119.0497 *m/z* from 193‐B.
**Figure S17.** Proposed formation mechanism of 150.0313 *m/z* and 122.0363 *m/z* from 179‐A.
**Figure S18.** Proposed formation mechanism of 117.0337 *m/z* and 135.0442 *m/z* with its product ion at 107.0491 *m/z* from 179‐A.

## Data Availability

The data that support the findings of this study are available from the corresponding author upon reasonable request.
