# Supplementary material for: Study of the Photoinduced Fate of Selected Contaminants in Surface Waters by HPLC‐HRMS
Source: Rapid Commun Mass Spectrom. 2025 May 20;39(17):e10075. doi: 10.1002/rcm.10075 (PMC12093083; doi:10.1002/rcm.10075)
Supplement: Supplementary file 1 — Figure S1. MS2 spectrum of epoxiconazole with its characteristic isotopic pattern. Table S1. MSn of epoxiconazole. Figure S2. MS2 spectrum of coumarin (top) and hymecromone (bottom). Table S2. MSn of coumarin (C9H7O2) and hymecromone (C10H9O3). Figure S3. Chromatographic separation of transformation products formed from photocatalysis of epoxiconazole. NL, normalized scale. Figure S4. Transformation products indicated as [MH]+ formed from epoxiconazole degradation as a function of the UV‐A light irradiation time in the presence of 400‐ppm TiO2. Figure S5. Isotopic pattern of intermediate 346 of epoxiconazole. Table S3. List of [MH]+ and fragments from MSn spectra with their empirical formulas and Δppm of (poly)hydroxylated TPs of epoxiconazole. Table S4. List of [MH]+ and fragments from MSn spectra with their empirical formulas and Δppm of dehalogenated TPs of epoxiconazole. Figure S6. Proposed formation mechanism of 310.0738 m/z and 259.0515 m/z with its product ion at 232.0569 m/z from intermediate 328‐A. Figure S7. Proposed formation mechanism of 216.0763 m/z, 188.0614 m/z with its product ion at 119.0490 m/z, and 154.9892 m/z from intermediate 344‐A. Table S5. List of [MH]+ and fragments from MSn spectra with their empirical formulas and Δppm of TPs from cleavage of epoxiconazole. Figure S8. Proposed formation mechanism of 179.0612 m/z, 151.0666 m/z, and 137.0397 m/z from intermediate 206. Figure S9. Isotopic pattern of intermediate 236 of epoxiconazole. Figure S10. Chromatographic separation of transformation products formed from coumarin. NL, normalized scale. Figure S11. Chromatographic separation of transformation products formed from hymecromone. NL, normalized scale. Figure S12. Transformation products indicated as [MH]+ formed from coumarin degradation as a function of the UV‐A light irradiation time in the presence of 400‐ppm TiO2. Figure S13. Transformation products indicated as [MH]+ formed from hymecromone degradation as a function of the UV‐A light i [file RCM-39-e10075-s001.docx]

**Study of the Photoinduced Fate of Selected Contaminants in Surface Waters by HPLC-HRMS**

Rossella Sesia^1^*, Federica Dal Bello^2^, Claudio Medana^2^, Rita Binetti^3^, Dimitra Papagiannaki^3^, Paola Calza^4^

1 Department of Management and Production Engineering, Politecnico di Torino, Italy

2 Department of Molecular Biotechnology and Health Sciences, University of Turin, Torino, Italy

3 SMAT, Torino, Italy

4 Department of Chemistry, University of Torino, Torino, Italy


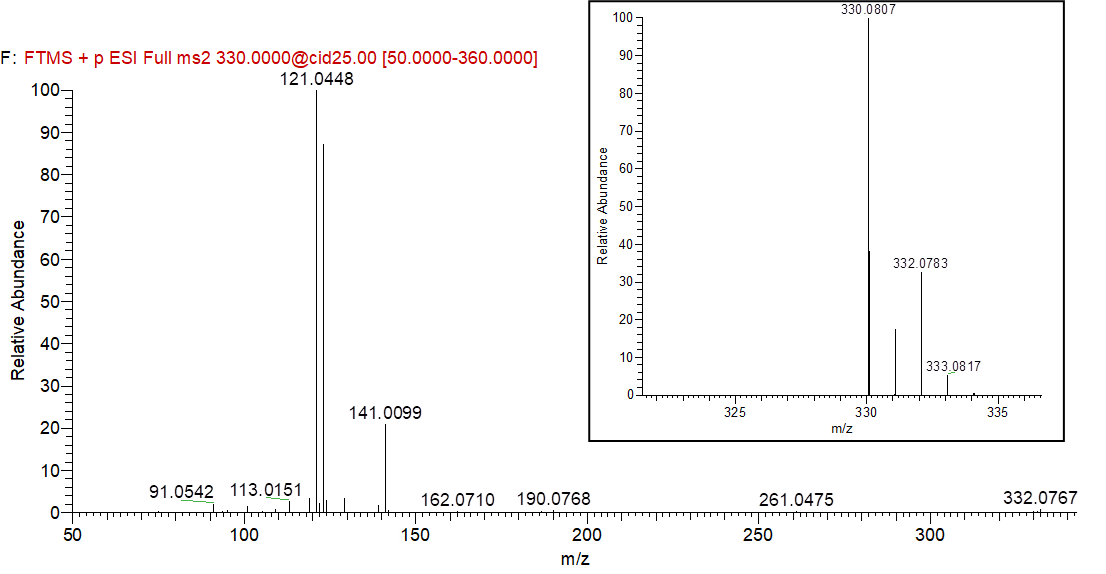


**Figure S1.** MS^2^ spectrum of epoxiconazole with its characteristic isotopic pattern.

**Table S1.** MS^n^ of epoxiconazole.

| **[MH]^+^ (*m/z*)** | **Empirical formula of [MH]^+^** | **MW** | **t_R_ (min)** | **MS^2^ (%)** | **Δppm** | **MS^3^ (%)** | **Δppm** |
| --- | --- | --- | --- | --- | --- | --- | --- |
| 330.0811 | C_17_H_14_ON_3_ClF | 329 | 23.38 | 312.0705 C_17_H_12_N_3_ClF  [<1] (-H_2_O) | 2.244 | - | - |
|  |  |  |  | 303.0702 C_16_H_13_N_2_OClF [<1]  (-HCN) | 2.193 | - | - |
|  |  |  |  | 261.0483 C_15_H_11_OClF  [1] (-C_2_H_3_N_3_) | -0.795 | 243.0382 C_15_H_9_ClF [1] (-H_2_O) | 4.473 |
|  |  |  |  |  |  | 165.0100 C_9_H_6_OCl [2] (-C_6_H_5_F) | -0.782 |
|  |  |  |  |  |  | 149.0399 C_9_H_6_OF [4] (-C_6_H_5_Cl) | 1.546 |
|  |  |  |  |  |  | 138.9944 C_7_H_4_OCl [45]  (-C_8_H_7_F) | -0.640 |
|  |  |  |  |  |  | 123.0240 C_7_H_4_OF [100]  (-C_8_H_7_Cl) | -1.703 |
|  |  |  |  |  |  | 121.0447 C_8_H_6_F [93]  (-C_7_H_5_OCl) | -1.446 |
|  |  |  |  | 190.0777 C_10_H_9_N_3_F [1]  (-C_7_H_5_OCl) | -3.641 | 121.0447 C_8_H_6_F [100]  (-C_2_H_3_N_3_) | 1.624 |
|  |  |  |  | 141.0101 C_7_H_6_ClO [20]  (-C_10_H_8_N_3_F) | -1.979 | 113.0150 C_6_H_6_Cl [23] (-CO) | -1.455 |
|  |  |  |  | 138.9944 C_7_H_4_OCl [2]  (-C_10_H_10_N_3_F) | -2.727 | - | - |
|  |  |  |  | 123.0240 C_7_H_4_OF [86]  (-C_10_H_10_ON_3_Cl) | -0.728 | - | - |
|  |  |  |  | 121.0447 C_8_H_6_F [100]  (-C_9_H_8_ON_3_Cl) | -0.289 | - | - |

**Figure S2.** MS^2^ spectrum of coumarin (top) and hymecromone (bottom).

**Table S2.** MS^n^ of coumarin (C_9_H_7_O_2_) and hymecromone (C_10_H_9_O_3_).

| **[MH]^+^ (*m/z*)** | **Empirical formula of [MH]^+^** | **MW** | **t_R_ (min)** | **MS^2^ (%)** | **Δppm** | **MS^3^ (%)** | **Δppm** |
| --- | --- | --- | --- | --- | --- | --- | --- |
| 147.0445 | C_9_H_7_O_2_ | 146 | 15.71 | 119.0496 C_8_H_7_O [1] (-CO) | 3.684 | 91.0543 C_7_H_7_ [84] (-CO) | -0.584 |
|  |  |  |  | 103.0549 C_8_H_7_ [100] (-CO_2_) | 6.144 | - | - |
|  |  |  |  | 91.0543 C_7_H_7_ [1] (-2CO) | 5.087 | - | - |
| 177.0551 | C_10_H_9_O_3_ | 176 | 14.54 | 149.0601 C_9_H_9_O_2_ [2] (-CO) | 1.110 | 121.0648 C_8_H_9_O [100] (-CO) | -0.177 |
|  |  |  |  | 133.0653 C_9_H_9_O [7] (-CO_2_) | 0.302 | 105.0699 C_8_H_9_ [100] (-CO) | 0.696 |
|  |  |  |  | 131.0496 C_9_H_7_O [<1] (-HCOOH) | 1.451 | - | - |
|  |  |  |  | 121.0648 C_8_H_9_O [6] (-2CO) | 0.166 | 93.0699 C_7_H_9_ [100] (-CO) | 0.463 |
|  |  |  |  | 105.0699 C_8_H_9_ [37] (-CO,-CO_2_) | 0.235 | - | - |
|  |  |  |  | 103.0547 C_8_H_7_ [3] (-C_2_H_2_O_3_) | -0.245 | - | - |
|  |  |  |  | 91.0547 C_7_H_7_ [<1] (-C_3_H_2_O_3_) | -0.606 | - | - |


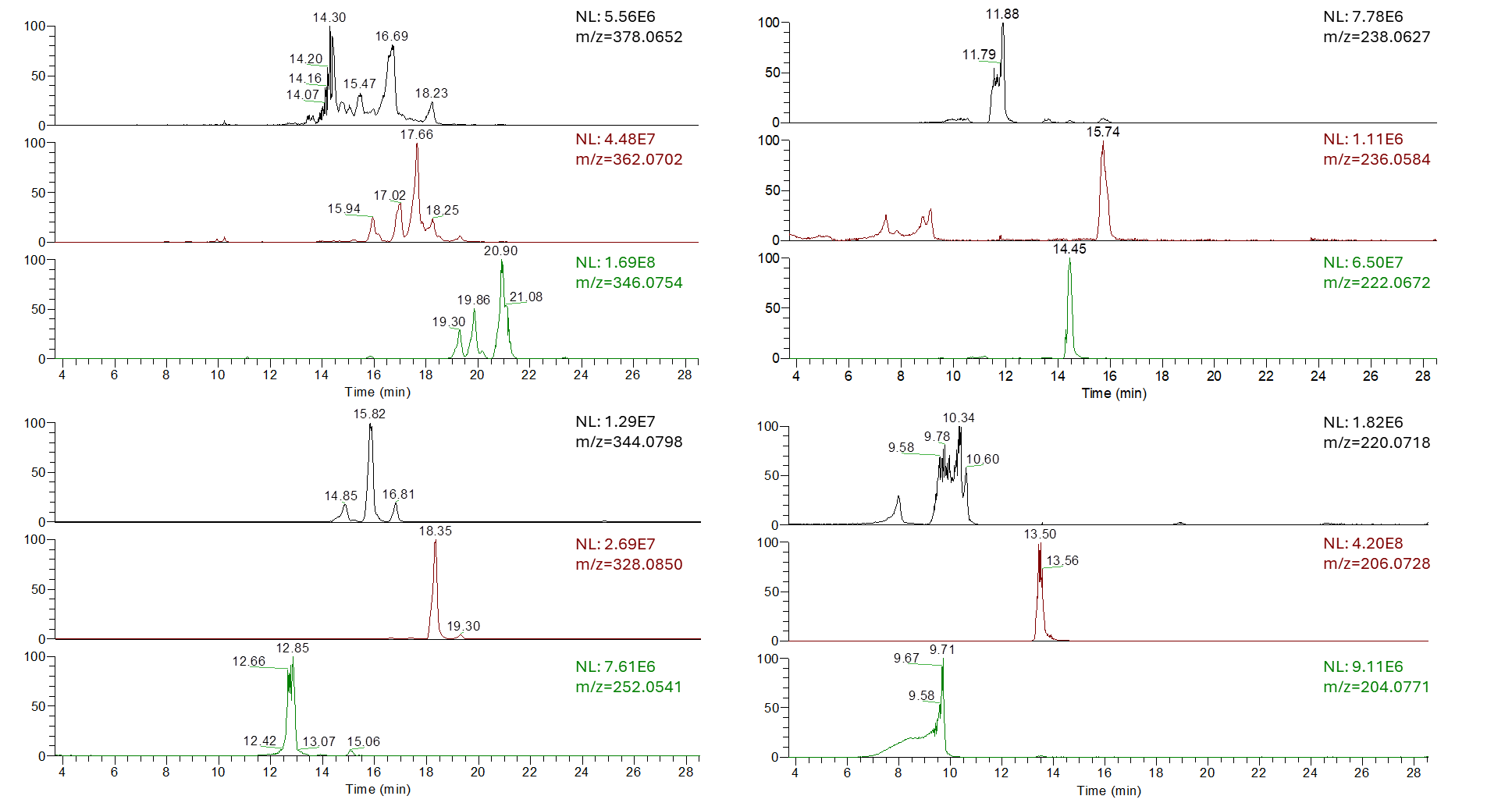


**Figure S3.** Chromatographic separation of transformation products formed from photocatalysis of epoxiconazole. NL, normalized scale


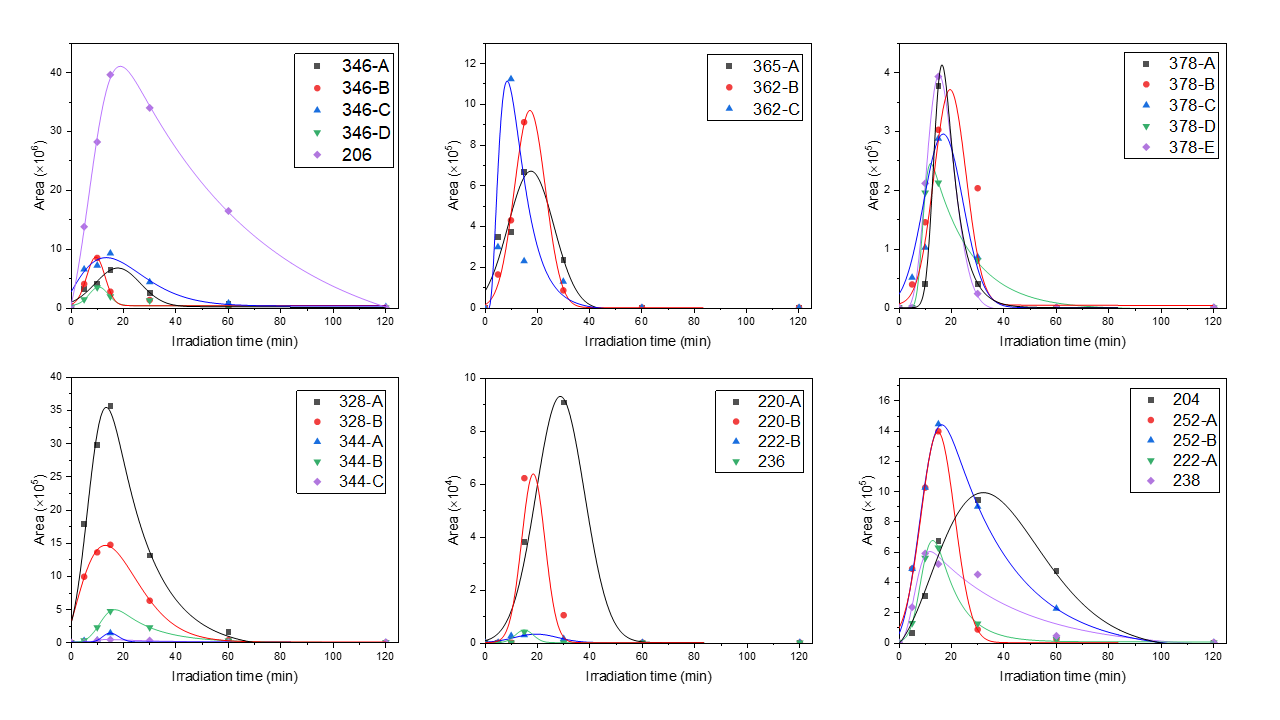


**Figure S4.** Transformation products indicated as [MH]^+^ formed from epoxiconazole degradation as a function of the UV-A light irradiation time in the presence of 400 ppm TiO_2_.


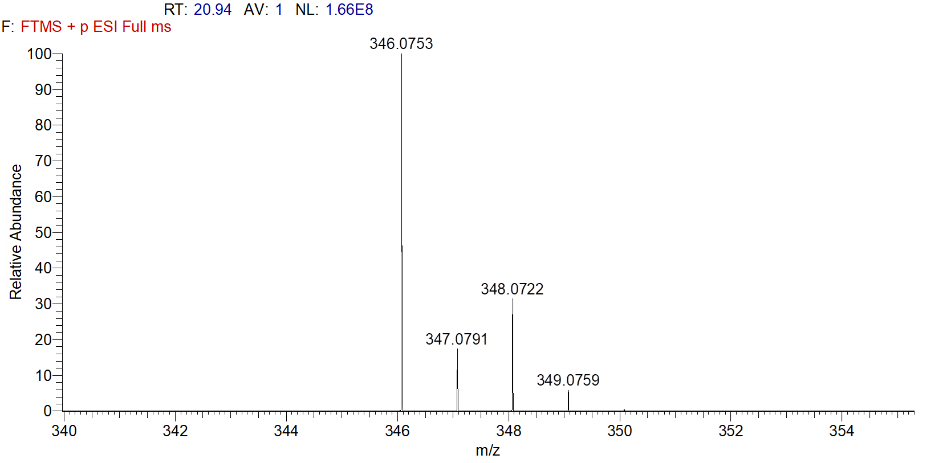


**Figure S5.** Isotopic pattern of intermediate 346 of epoxiconazole.

**Table S3.** List of [MH]^+^ and fragments from MS^n^ spectra with their empirical formulas and Δppm of (poly)hydroxylated TPs of epoxiconazole.

| **[MH]^+^ (*m/z*)** | **Empirical formula of [MH]^+^** | **MW** | **t_R_ (min)** | **MS^2^ (%)** | **Δppm** | **MS^3^ (%)** | **Δppm** |
| --- | --- | --- | --- | --- | --- | --- | --- |
| 346.0756  346-A | C_17_H_14_O_2_N_3_ClF | 345-A | 19.30 | 277.0433 C_15_H_11_O_2_ClF [10] (-C_2_H_3_N_3_) | 2.375 | - | - |
|  |  |  |  | 259.0327 C_15_H_9_OClF [17] (-C_2_H_5_ON_3_) | 2.558 | - | - |
|  |  |  |  | 224.0637 C_15_H_9_OF [2] (-C_2_H_5_ON_3_Cl) | 2.478 | - | - |
|  |  |  |  | 141.0101 C_7_H_6_OCl [28] (-C_10_H_8_ON_3_F) | -0.073 | - | - |
|  |  |  |  | 138.9944 C_7_H_4_OCl [100] (-C_10_H_10_ON_3_F) | -0.497 | - | - |
| 346.0753  346-B |  | 345-B | 19.86 | 290.0387 C_15_H_10_O_2_NClF [3] (-C_2_H_4_N_2_) | 2.789 | - | - |
|  |  |  |  | 277.0433 C_15_H_11_O_2_ClF [12] (-C_2_H_3_N_3_) | 2.267 | - | - |
|  |  |  |  | 190.0779 C_10_H_9_N_3_F [3] (-C_7_H_5_O_2_Cl) | -0.581 | - | - |
|  |  |  |  | 157.0052 C_7_H_6_O_2_Cl [26] (-C_10_H_8_N_3_F) | 0.741 | - | - |
|  |  |  |  | 123.0239 C_7_H_4_OF [93] (-C_10_H_10_O_2_N_3_Cl) | -1.134 | - | - |
|  |  |  |  | 121.0447 C_8_H_6_F [100] (-C_9_H_8_O_2_N_3_Cl) | -1.198 | - | - |
| 346.0753  346-C |  | 345-C | 20.90 | 328.0655 C_17_H_12_ON_3_ClF [6] (-H_2_O) | 2.364 | - | - |
|  |  |  |  | 290.0387 C_15_H_10_O_2_NClF [6] (-C_2_H_4_N_2_) | 2.686 | - | - |
|  |  |  |  | 277.0433 C_15_H_11_O_2_ClF [4] (-C_2_H_3_N_3_) | 2.686 | 149.0395 C_9_H_6_OF [99] (-C_6_H_5_OCl) | -5.019 |
|  |  |  |  | 259.0328 C_15_H_9_OClF [8] (-C_2_H_5_ON_3_) | 2.944 | - | - |
|  |  |  |  | 190.0779 C_10_H_9_N_3_F [3] (-C_7_H_5_O_2_Cl) | -4.918 | - | - |
|  |  |  |  | 157.0052 C_7_H_6_O_2_Cl [37] (-C_10_H_8_N_3_F) | 0.741 | - | - |
|  |  |  |  | 123.0239 C_7_H_4_OF [100] (-C_10_H_10_ON_3_Cl) | 1.134 | - | - |
|  |  |  |  | 121.0447 C_8_H_6_F [94] (-C_9_H_8_O_2_N_3_Cl) | -1.033 | - | - |
| 346.0754  346-D |  | 345-D | 21.08 | 328.0655 C_17_H_12_ON_3_ClF [13] (-H_2_O) | 2.273 | - | - |
|  |  |  |  | 290.0386 C_15_H_10_O_2_NClF [7] (-C_2_H_4_N_2_) | 2.445 | - | - |
|  |  |  |  | 277.0433 C_15_H_11_O_2_ClF [6] (-C_2_H_3_N_3_) | 2.750 | 123,0241 C_7_H_4_OF [100] (-C_8_H_7_OCl) | -3,478 |
|  |  |  |  | 259,0327 C_15_H_9_OClF [100] (-C_2_H_5_ON_3_) | 2,713 | - | - |
|  |  |  |  | 224.06230 C_15_H_9_OF [4] (-C_2_H_5_ON_3_Cl) | -0.913 | - | - |
|  |  |  |  | 141.0101 C_7_H_6_OCl [30] (-C_10_H_8_ON_3_F) | -0.135 | - | - |
| 362.0705  362-A | C_17_H_14_O_3_N_3_ClF | 361-A | 15.94 | 293.0377 C_15_H_11_O_3_ClF [<1] (-C_2_H_3_N_3_) | -1.416 | 141.0101 C_7_H_6_ClO [66] (-C_8_H_5_O_2_F) | -3.315 |
|  |  |  |  |  |  | 138.9944 C_7_H_4_OCl [100] (-C_8_H_7_O_2_F) | -4.154 |
|  |  |  |  | 234,0428 C_11_H_9_ON_3_Cl [<1] (-C_6_H_5_O_2_F) | -2.754 | - | - |
|  |  |  |  | 222.0672 C_10_H_9_O_2_N_3_F [<1] (-C_7_H_5_OCl) | -2.296 | - | - |
|  |  |  |  | 138.9944 C_7_H_4_OCl [100] (-C_10_H_10_O_2_F) | -4.154 | - | - |
| 362.0702  362-B |  | 361-B | 17.02 | 250.0376 C_11_H_9_O_2_N_3_Cl [<1] (-C_6_H_5_OF) | -2.916 |  |  |
|  |  |  |  | 149.0398 C_9_H_6_OF [100] (-C_8_H_8_O_2_N_3_Cl) | 2.872 | - | - |
|  |  |  |  | 141.0101 C_7_H_6_OCl [81] (-C_10_H_8_O_2_N_3_) | -4.024 | - | - |
|  |  |  |  | 125.0398 C_7_H_6_OF [21] (-C_10_H_8_O_2_N_3_Cl) | -5.982 | - | - |
|  |  |  |  | 113.0153 C_6_H_6_Cl [21] (-C_11_H_8_O_3_N_3_F) | -4.096 | - | - |
| 362.0703  362-C |  | 361-C | 17.66 | 293.0377 C_15_H_11_O_3_ClF [<1] (-C_2_H_3_N_3_) | -3.600 | 125.0397 C_7_H_6_OF [77] (-C_8_H_5_O_2_Cl) | -4.462 |
|  |  |  |  |  |  | 123.0241 C_7_H_4_OF [100] (-C_8_H_7_O_2_Cl) | -3.885 |
|  |  |  |  |  |  | 121.0447 C_8_H_6_F [14] (-C_9_H_8_O_3_N_3_Cl) | -6.059 |
|  |  |  |  |  |  | 97.0448 C_6_H_6_F [21] (-C_9_H_5_O_3_Cl) | -5.908 |
|  |  |  |  | 154.9892 C_7_H_4_O_2_Cl [28] (-C_10_H_10_ON_3_F) | -5.368 | - | - |
|  |  |  |  | 125.0398 C_7_H_6_OF [86] (-C_10_H_8_O_2_N_3_Cl) | -3.503 | - | - |
|  |  |  |  | 123.0241 C_7_H_4_OF [100] (-C_10_H_10_O_2_N_3_Cl) | -4.210 | - | - |
|  |  |  |  | 121.0447 C_8_H_6_F [27] (-C_9_H_8_O_3_N_3_Cl) | -5.563 | - | - |
| 378.0652  378-A | C_17_H_14_O_4_N_3_ClF | 377-A | 14.30 | 291.0215 C_15_H_9_O_3_ClF [10] (-C_2_H_5_ON_3_) | -4.072 | - | - |
|  |  |  |  | 206.0719 C_10_H_9_ON_3_F [14] (-C_7_H_5_O_3_Cl) | -5.120 | - | - |
|  |  |  |  | 165.0344 C_9_H_6_O_2_F [6] (-C_8_H_8_O_2_N_3_Cl) | -4.681 | - | - |
|  |  |  |  | 157.0049 C_7_H_6_O_2_Cl [74] (-C_10_H_8_O_2_N_3_F) | -5.045 | - | - |
|  |  |  |  | 154.9892 C_7_H_4_O_2_Cl [100] (-C_10_H_10_O_2_N_3_F) | -5.433 | - | - |
|  |  |  |  | 125.0396 C_7_H_6_OF [40] (-C_10_H_8_O_3_N_3_Cl) | -5.262 | - | - |
| 378.0652  378-B |  | 377-B | 14.75 | 291.0212 C_15_H_9_O_3_ClF [27] (-C_2_H_5_ON_3_) | -4.278 | - | - |
|  |  |  |  | 165.0350 C_9_H_6_O_2_F [11] (-C_8_H_8_O_2_N_3_Cl) | -5.467 | - | - |
|  |  |  |  | 157.0047 C_7_H_6_O_2_Cl [22] (-C_10_H_8_O_2_N_3_F) | -6.128 | - | - |
|  |  |  |  | 141.0344 C_7_H_6_O_2_F [100] (-C_10_H_8_O_2_N_3_Cl) | -5.478 | - | - |
| 378.0653  378-C |  | 377-C | 15.47 | 293.0366 C_15_H_11_O_3_ClF [12] (-C_2_H_3_ON_3_) | -5.068 | - | - |
|  |  |  |  | 265.0419 C_14_H_11_O_2_ClF [19] (-C_3_H_3_O_2_N_3_) | -4.869 | - | - |
|  |  |  |  | 229.0654 C_14_H_10_O_2_F [24] (-C_3_H_4_O_2_N_3_Cl) | -4.552 | - | - |
|  |  |  |  | 206.0719 C_10_H_9_ON_3_F [48] (-C_7_H_5_O_3_Cl) | -5.848 | - | - |
|  |  |  |  | 160.9997 C_6_H_6_O_3_Cl [44] (-C_11_H_8_ON_3_F) | -5.010 | - | - |
|  |  |  |  | 151.0550 C_9_H_8_OF [23] (-C_8_H_6_O_3_N_3_Cl) | -6.210 | - | - |
|  |  |  |  | 142.9892 C_6_H_4_O_2_Cl [47] (-C_11_H_10_O_2_N_3_F) | -5.609 | - | - |
|  |  |  |  | 133.0049 C_5_H_6_O_2_Cl [61] (-C_12_H_8_O_2_N_3_F) | -5.655 | - | - |
|  |  |  |  | 123.0239 C_7_H_4_OF [100] (-C_10_H_10_O_3_N_3_Cl) | -5.754 | - | - |
| 378.0652  378-D |  | 377-D | 16.69 | 309.0320 C_15_H_11_O_4_ClF [16] (-C_2_H_3_N_3_) | -3.073 | - | - |
|  |  |  |  | 291.0215 C_15_H_9_O_3_ClF [9] (-C_2_H_5_ON_3_) | -3.350 | - | - |
|  |  |  |  | 273.0550 C_15_H_10_O_4_F [6] (-C_2_H_4_N_3_Cl) | -4.731 | - | - |
|  |  |  |  | 263.0265 C_14_H_9_O_2_ClF [5] (-C_3_H_5_O_2_N_3_) | -3.765 | - | - |
|  |  |  |  | 243.0448 C_14_H_8_O_3_F [<1] (-C_3_H_6_ON_3_Cl) | -3.774 | - | - |
|  |  |  |  | 227.0500 C_14_H_8_O_2_F [15] (-C_3_H_6_O_2_N_3_Cl) | -3.447 | - | - |
|  |  |  |  | 206.0721 C_10_H_9_ON_3_F [49] (-C_7_H_5_O_3_Cl) | -4.198 | - | - |
|  |  |  |  | 157.0047 C_7_H_6_O_2_Cl [<1] (-C_10_H_8_O_2_N_3_F) | -4.090 | - | - |
|  |  |  |  | 125.0396 C_7_H_6_OF [10] (-C_10_H_8_O_3_N_3_Cl) | -5.742 | - | - |
|  |  |  |  | 123.0239 C_7_H_4_OF [35] (-C_10_H_10_O_3_N_3_Cl) | -5.510 | - | - |
|  |  |  |  | 121.0447 C_8_H_6_F [100] (-C_9_H_8_O_4_N_3_Cl) | -4.895 | - | - |
|  |  |  |  | 109.0448 C_7_H_6_F [11] (-C_10_H_8_O_4_N_3_Cl) | -5.258 | - | - |
| 378.0654  378-E |  | 377-E | 18.23 | 291.0212 C_15_H_9_O_3_ClF [27] (-C_2_H_5_ON_3_) | -4.278 | - | - |
|  |  |  |  | 271.0392 C_14_H_8_O_4_F [1] (-C_3_H_6_N_3_Cl) | -5.467 | - | - |
|  |  |  |  | 227.0497 C_14_H_8_O_2_F [46] (-C_3_H_6_O_2_N_3_Cl) | -5.077 | - | - |
|  |  |  |  | 206.0719 C_10_H_9_ON_3_F [100] (-C_7_H_5_O_3_Cl) | -4.926 | - | - |
|  |  |  |  | 123.0239 C_7_H_4_OF [35] (-C_10_H_10_O_3_N_3_Cl) | -1.053 | - | - |
|  |  |  |  | 121.0447 C_8_H_6_F [83] (-C_9_H_8_O_4_N_3_Cl) | -5.563 | - | - |

**Table S4.** List of [MH]^+^ and fragments from MS^n^ spectra with their empirical formulas and Δppm of dehalogenated TPs of epoxiconazole.

| **[MH]^+^ (*m/z*)** | **Empirical formula of [MH]^+^** | **MW** | **t_R_ (min)** | **MS^2^ (%)** | **Δppm** | **MS^3^ (%)** | **Δppm** |
| --- | --- | --- | --- | --- | --- | --- | --- |
| 328.0850  328-A | C_17_H_15_O_2_N_3_Cl | 327-A | 18.35 | 310.0738 C_17_H_13_ON_3_Cl [1] (-H_2_O) | -1.214 | - | - |
|  |  |  |  | 259.0515 C_15_H_12_O_2_Cl [1] (-C_2_H_3_N_3_) | -1.906 | - | - |
|  |  |  |  | 231.0569 C_14_H_12_OCl [1] (-C_3_H_3_ON_3_) | -0.861 | - | - |
|  |  |  |  | 188.0815 C_10_H_10_ON_3_ [8] (-C_7_H_5_OCl) | -1.800 | 119.0492 C_9_H_7_O_2_ [100] (-C_8_H_8_N_3_Cl) | -1.452 |
|  |  |  |  | 147.0440 C_9_H_7_O_2_ [2] (-C_8_H_8_N_3_Cl) | -0.517 | - | - |
|  |  |  |  | 138.9944 C_7_H_4_OCl [21] (-C_10_H_11_ON_3_) | -0.856 | - | - |
|  |  |  |  | 121.0284 C_7_H_5_O_2_ [64] (-C_10_H_10_N_3_Cl) | -0.049 | - | - |
|  |  |  |  | 119.0492 C_8_H_7_O [100] (-C_9_H_8_ON_3_Cl) | 0.492 | - | - |
|  |  |  |  | 107.0491 C_7_H_7_O [2] (-C_10_H_8_ON_3_Cl) | -3.828 | - | - |
| 328.0850 328-B |  | 327-B | 19.30 | 188.0815 C_10_H_10_ON_3_ [2] (-C_7_H_5_OCl) | -4.716 | 119.0490 C_9_H_7_O_2_ [100] (-C_8_H_8_N_3_Cl) | -3.957 |
|  |  |  |  | 147.0437 C_9_H_7_O_2_ [2] (-C_8_H_8_N_3_Cl) | -6.151 | - | - |
|  |  |  |  | 138.9944 C_7_H_4_OCl [21] (-C_10_H_11_ON_3_) | -5.162 | - | - |
|  |  |  |  | 121.0283 C_7_H_5_O_2_ [37] (-C_10_H_10_N_3_Cl) | -5.076 | - | - |
|  |  |  |  | 119.0491 C_8_H_7_O [100] (-C_9_H_8_ON_3_Cl) | -4.954 | - | - |
| 344.0797  344-A | C_17_H_15_O_3_N_3_Cl | 343-A | 14.85 | 216.0763 C_11_H_10_O_2_N_3_ClF [4] (-C_6_H_5_OCl) | -4.866 | - | - |
|  |  |  |  | 188.0814 C_10_H_10_ON_3_ [6] (-C_7_H_5_O_2_Cl) | -5.300 | - | - |
|  |  |  |  | 154.9892 C_7_H_4_O_2_Cl [13] (-C_10_H_11_ON_3_) | -4.852 | - | - |
|  |  |  |  | 147.0438 C_9_H_7_O_2_ [28] (-C_8_H_8_ON_3_Cl) | -5.199 | - | - |
|  |  |  |  | 121.0283 C_7_H_5_O_2_ [57] (-C_10_H_10_ON_3_Cl) | -5.241 | - | - |
|  |  |  |  | 119.0490 C_8_H_7_O [100] (-C_9_H_8_O_2_N_3_Cl) | -5.458 | - | - |
|  |  |  |  | 107.0491 C_7_H_7_O [9] (-C_10_H_8_O_2_N_3_Cl) | -5.697 | - | - |
| 344.0799  344-B |  | 343-B | 15.82 | 326.0677 C_17_H_13_O_2_N_3_Cl [1] (-H_2_O) | -5.586 | - | - |
|  |  |  |  | 257.0359 C_15_H_10_O_2_Cl [68] (-C_2_H_5_ON_3_) | -4.210 | 222.0672 C_15_H_10_O_2_ [82] (-Cl) | -4.051 |
|  |  |  |  | 234.0424 C_11_H_9_ON_3_Cl [44] (-C_6_H_6_O_2_) | -4.549 | 165.0103 C_9_H_6_OCl [39] (-C_2_H_3_N_3_) | -2.348 |
|  |  |  |  |  |  | 137.0152 C_8_H_6_Cl [100] (-C_3_H_3_ON_3_) | -4.546 |
|  |  |  |  | 222.0672 C_15_H_10_O_2_ [5] (-C_2_H_5_ON_3_Cl) | -4.096 | - | - |
|  |  |  |  | 188.0814 C_10_H_10_ON_3_ [6] (-C_7_H_5_O_2_Cl) | -5.460 | - | - |
|  |  |  |  | 121.0283 C_7_H_5_O_2_ [62] (-C_10_H_10_ON_3_Cl) | -5.159 | - | - |
|  |  |  |  | 119.0490 C_8_H_7_O [100] (-C_9_H_8_O_2_N_3_Cl) | -5.459 | - | - |
|  |  |  |  | 107.0491 C_7_H_7_O [27] (-C_10_H_8_O_2_N_3_Cl) | -5.229 | - | - |
| 344.0798  344-C |  | 343-C | 16.84 | 163.0387 C_9_H_7_O_3_ [5] (-C_8_H_8_N_3_Cl) | -4.962 | - | - |
|  |  |  |  | 141.0100 C_7_H_6_OCl [13] (-C_10_H_10_N_3_Cl) | -4.166 | - | - |
|  |  |  |  | 137.0232 C_7_H_5_O_3_ [55] (-C_10_H_10_N_3_Cl) | -4.809 | - | - |
|  |  |  |  | 135.0440 C_8_H_7_O_2_ [100] (-C_9_H_8_ON_3_Cl) | -4.772 | - | - |
|  |  |  |  | 125.0231 C_6_H_5_O_3_ [4] (-C_11_H_10_N_3_Cl) | -5.751 | - | - |


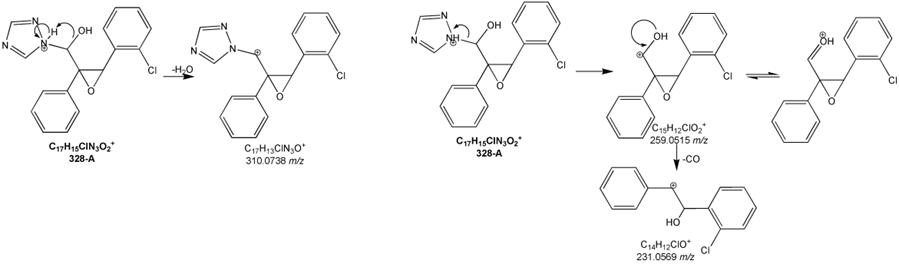


**Figure S6.** Proposed formation mechanism of 310.0738 *m/z* and 259.0515 *m/z* with its product ion at 232.0569 *m/z* from intermediate 328-A.


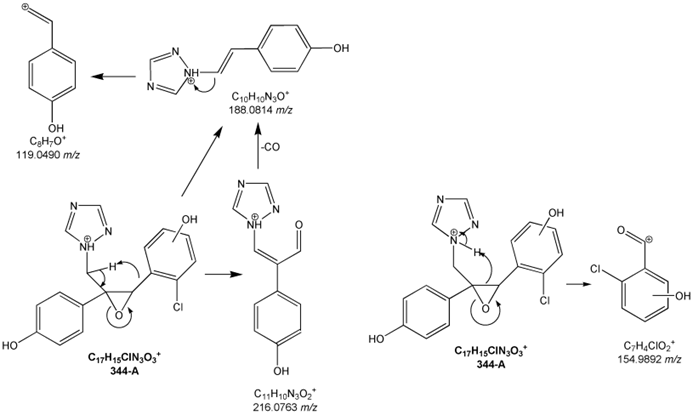


**Figure S7.** Proposed formation mechanism of 216.0763 *m/z*, 188.0614 *m/z* with its product ion at 119.0490 *m/z*, and 154.9892 *m/z* from intermediate 344-A.

**Table S5.** List of [MH]^+^ and fragments from MS^n^ spectra with their empirical formulas and Δppm of TPs from cleavage of epoxiconazole.

| **[MH]^+^ (*m/z*)** | **Empirical formula of [MH]^+^** | **MW** | **t_R_ (min)** | **MS^2^ (%)** | **Δppm** | **MS^3^ (%)** | **Δppm** |
| --- | --- | --- | --- | --- | --- | --- | --- |
| 206.0728 | C_10_H_9_ON_3_F | 205 | 13.50 | 179.0612 C_9_H_8_ON_2_F [16] (-HCN) | -4.837 | 151.0666 C_8_H_8_N_2_F [13] (-CO) | -0.285 |
|  |  |  |  |  |  | 137.0397 C_8_H_6_OF [63] (-CH_2_N_2_) | -3.999 |
|  |  |  |  |  |  | 136.0557 C_8_H_7_NF [100] (-CHON) | -3.987 |
|  |  |  |  |  |  | 109.0448 C_7_H_6_F [8] (-C_2_H_2_ON_2_) | -4.708 |
|  |  |  |  | 151.0664 C_8_H_8_N_2_F [9] (-C_2_HON) | -4.908 | - | - |
|  |  |  |  | 137.0397 C_8_H_6_OF [43] (-C_2_H_3_N_3_) | -4.437 | 109.0449 C_7_H_6_F [100] (-CO) | -4.066 |
|  |  |  |  | 136.0556 C_8_H_7_NF [41] (-C_2_H_2_ON_2_) | -4.428 | - | - |
|  |  |  |  | 123.0240 C_7_H_4_OF [<1] (-C_3_H_5_N_3_) | -5.023 | - | - |
|  |  |  |  | 109.0448 C_7_H_6_F [24] (-C_3_H_3_ON_3_) | -5.167 | - | - |
| 204.0771 | C_10_H_10_O_2_N_3_ | 203 | 9.70 | 177.0655 C_9_H_9_O_2_N_2_ [1] (-HCN) | -5.210 | 149.0706 C_8_H_9_ON_2_ [100] (-CO) | -4.776 |
|  |  |  |  | 149.0706 C_8_H_9_ON_2_ [2] (-C_2_HON) | -6.023 | - | - |
|  |  |  |  | 135.0440 C_8_H_7_O_2_ [67] (-C_2_H_3_N_3_) | -5.023 | 107.0493 C_7_H_7_O [100] (-CO) | -3.523 |
|  |  |  |  | 107.0493 C_7_H_7_O [100] (-C_3_H_3_ON_3_) | -3.735 | - | - |
| 220.0716  220-A | C_10_H_10_O_3_N_3_ | 219-A | 8.00 | 151.0387 C_8_H_7_O_3_ [23] (-C_2_H_3_N_3_) | -5.224 | 123.0440 C_7_H_7_O_2_ [100] (-C_3_H_3_ON_3_) | -4.007 |
|  |  |  |  | 123.0439 C_7_H_7_O_2_ [100] (-C_3_H_3_ON_3_) | -5.319 | 95.0491 C_6_H_7_O [100] (-CO) | -5.224 |
|  |  |  |  | 95.0491 C_6_H_7_O [1] (-C_4_H_3_O_2_N_3_) | -6.521 | - | - |
|  |  |  |  | 77.0385 C_6_H_5_ [5] (-C_4_H_5_O_3_N_3_) | -7.725 | - | - |
|  |  |  |  | 70.0399 C_2_H_4_N_3_ [38] (-C_8_H_6_O_3_) | -9.025 | - | - |
| 220.0718  220-B |  | 219-B | 10.34 | 151.0388 C_8_H_7_O_3_ [21] (-C_2_H_3_N_3_) | -5.091 | 123.0439 C_7_H_7_O_2_ [100] (-CO) | -5.096 |
|  |  |  |  | 123.0439 C_7_H_7_O_2_ [100] (-C_3_H_3_ON_3_) | -5.319 | 95.0491 C_6_H_7_O [89] (-CO) | -5.837 |
|  |  |  |  | 111.0440 C_6_H_7_O_2_ [7] (-C_4_H_3_ON_3_) | -5.623 | - | - |
|  |  |  |  | 95.0491 C_6_H_7_O [5] (-C_4_H_3_O_2_N_3_) | -6.100 | - | - |
|  |  |  |  | 82.0399 C_3_H_4_N_3_ [1] (-C_7_H_6_O_3_) | -6.974 | - | - |
|  |  |  |  | 70.0400 C_2_H_4_N_3_ [4] (-C_8_H_6_O_3_) | -6.455 | - | - |
| 222.0672  222-A | C_10_H_9_O_2_N_3_F | 221-A | 14.30 | 153.0347 C_8_H_6_O_2_F [28] (-C_2_H_3_N_3_) | -4.253 | 123.0240 C_7_H_4_OF [100] (-C_3_H_3_ON_3_) | -3.997 |
|  |  |  |  | 137.0390 C_8_H_6_FO [2] (-C_2_H_3_ON_3_) | -5.019 | 109.0448 C_7_H_6_F [94] (-CO) | -3.003 |
|  |  |  |  | 123.0241 C_7_H_4_OF [100] (-C_3_H_5_ON_3_) | -5.319 | - | - |
| 222.0672  222-B |  | 221-B | 14.50 | 153.0347 C_8_H_6_O_2_F [18] (-C_2_H_3_N_3_) | -4.246 | 123.0240 C_7_H_4_OF [100] (-C_3_H_3_ON_3_) | -3.858 |
|  |  |  |  | 139.0188 C_7_H_4_O_2_F [100] (-C_3_H_5_N_3_) | -4.079 | 111.0241 C_6_H_4_OF [83] (-CO) | -4.054 |
|  |  |  |  | 125.0397 C_7_H_6_OF [22] (-C_3_H_3_ON_3_) | -4.003 | - | - |
| 238.0627 | C_10_H_9_O_3_N_3_F | 237 | 11.88 | 211.0509 C_9_H_8_O_3_N_2_F (-HCN) | -4.763 | - | - |
|  |  |  |  | 169.0293 C_8_H_6_O_3_F [26] (-C_2_H_3_N_3_) | -4.894 | 141.0343 C_7_H_6_O_2_F [100] (-CO) | -4.008 |
|  |  |  |  | 141.0345 C_7_H_6_O_2_F [54] (-C_3_H_3_ON_3_) | -4.982 | 113.0397 C_6_H_6_OF [100] (-CO) | -5.230 |
|  |  |  |  | 113.0397 C_6_H_6_OF [29] (-C_4_H_3_O_2_N_3_) | -5.290 | - | - |
|  |  |  |  | 70.0401 C_2_H_4_N_3_ [97] (-C_2_H_4_N_3_) | -6.024 | - | - |
| 236.0583 | C_11_H_11_ON_3_OCl | 235 | 15.74 | 167.0260 C_9_H_8_OCl [15] (-C_2_H_3_N_3_) | -3.976 | 138.9944 C_7_H_4_OCl [100] (-C_2_H_4_) | -2.855 |
|  |  |  |  | 153.0102 C_8_H_6_OCl [38] (-C_3_H_5_N_3_) | -4.558 | 125.0153 C_7_H_6_Cl [100] (-CO) | -2.763 |
|  |  |  |  | 138.9944 C_7_H_4_OCl [100] (-C_4_H_7_N_3_) | -3.252 | - | - |
|  |  |  |  | 70.0401 C_2_H_4_N_3_ (-C_9_H_7_OCl) | -4.738 | - | - |
| 252.0540  252-A | C_11_H_11_O_2_N_3_Cl | 251-A | 12.87 | 234.0424 C_11_H_9_ON_3_Cl [1] (-H_2_O) | -4.378 | 138.9944 C_7_H_4_OCl [43] (-C_4_H_5_N_3_) | -4.855 |
|  |  |  |  | 183.0204 C_9_H_8_O_2_Cl [100] (-C_2_H_3_N_3_) | -4.711 | 165.0097 C_9_H_6_OCl [96] (-H_2_O) | -4.943 |
|  |  |  |  | 165.0097 C_9_H_6_OCl [2] (-C_3_H_5_ON_3_) | -5.924 | - | - |
|  |  |  |  | 155.0256 C_8_H_8_OCl [35] (-C_3_H_3_ON_3_) | -5.145 | 137.0151 C_8_H_6_Cl [49] (-H_2_O) | -4.407 |
|  |  |  |  |  |  | 113.0153 C_6_H_6_Cl [17] (-CO) | -4.002 |
|  |  |  |  | 148.0517 C_9_H_8_O_2_ [1] (C_2_H_3_N_3_Cl) | -5.145 | - | - |
|  |  |  |  | 138.9944 C_7_H_4_OCl [43] (-C_4_H_7_ON_3_) | -5.162 | - | - |
|  |  |  |  | 137.0151 C_8_H_6_Cl [26] (-C_3_H_5_O_2_N_3_) | -5.203 | - | - |
|  |  |  |  | 119.0491 C_8_H_7_O [39] (-C_3_H_4_ON_3_Cl) | -5.290 | - | - |
|  |  |  |  | 113.0153 C_6_H_6_Cl [4] (-C_5_H_5_O_2_N_3_) | -5.688 | - | - |
|  |  |  |  | 91.0542 C_7_H_7_ [17] (-C_4_H_4_O_2_N_3_Cl) | -5.878 | - | - |
| 252.0540  252-B |  | 251-B | 15.06 | 183.0202 C_9_H_8_O_2_Cl [7] (-C_2_H_3_N_3_) | -6.254 | 165.0098 C_9_H_6_OCl [100] (-H_2_O) | -5.086 |
|  |  |  |  | 165.0098 C_9_H_6_OCl [100] (-C_3_H_5_ON_3_) | -5.560 | - | - |
|  |  |  |  | 110..0348 C_4_H_4_ON_3_ [46] (C_7_H_7_OCl) | -5.696 | - | - |
|  |  |  |  | 154..9888 C_7_H_4_O_2_Cl [<1] (-C_4_H_7_N_3_) | -7.304 | - | - |


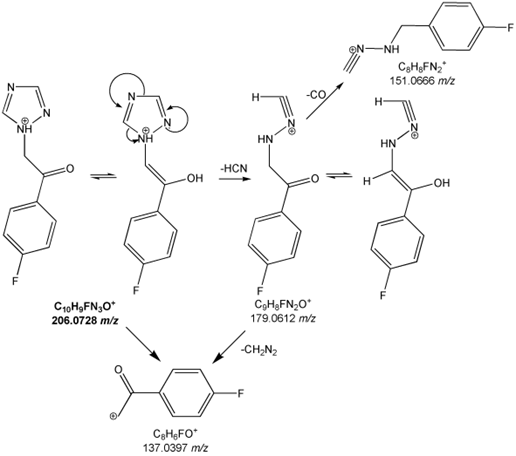


**Figure S8.** Proposed formation mechanism of 179.0612 *m/z*, 151.0666 *m/z*, and 137.0397 *m/z* from intermediate 206.


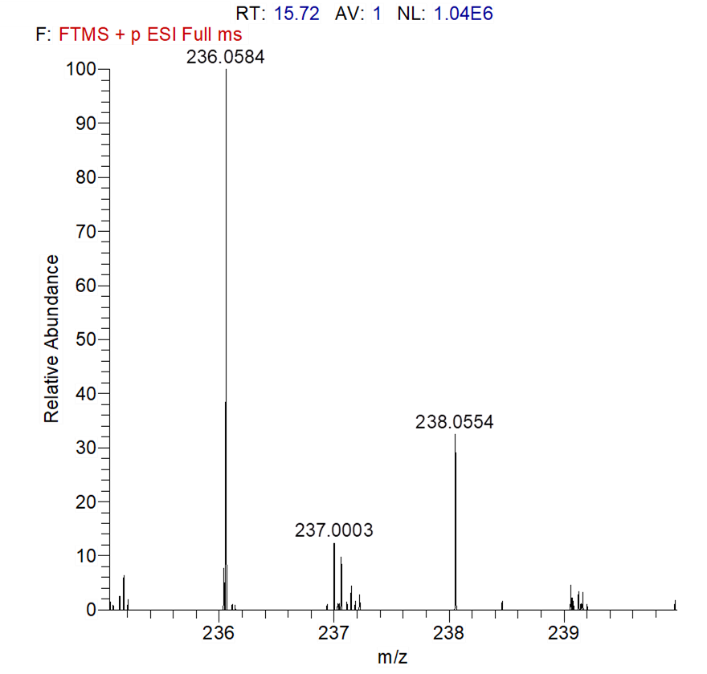


**Figure S9.** Isotopic pattern of intermediate 236 of epoxiconazole.


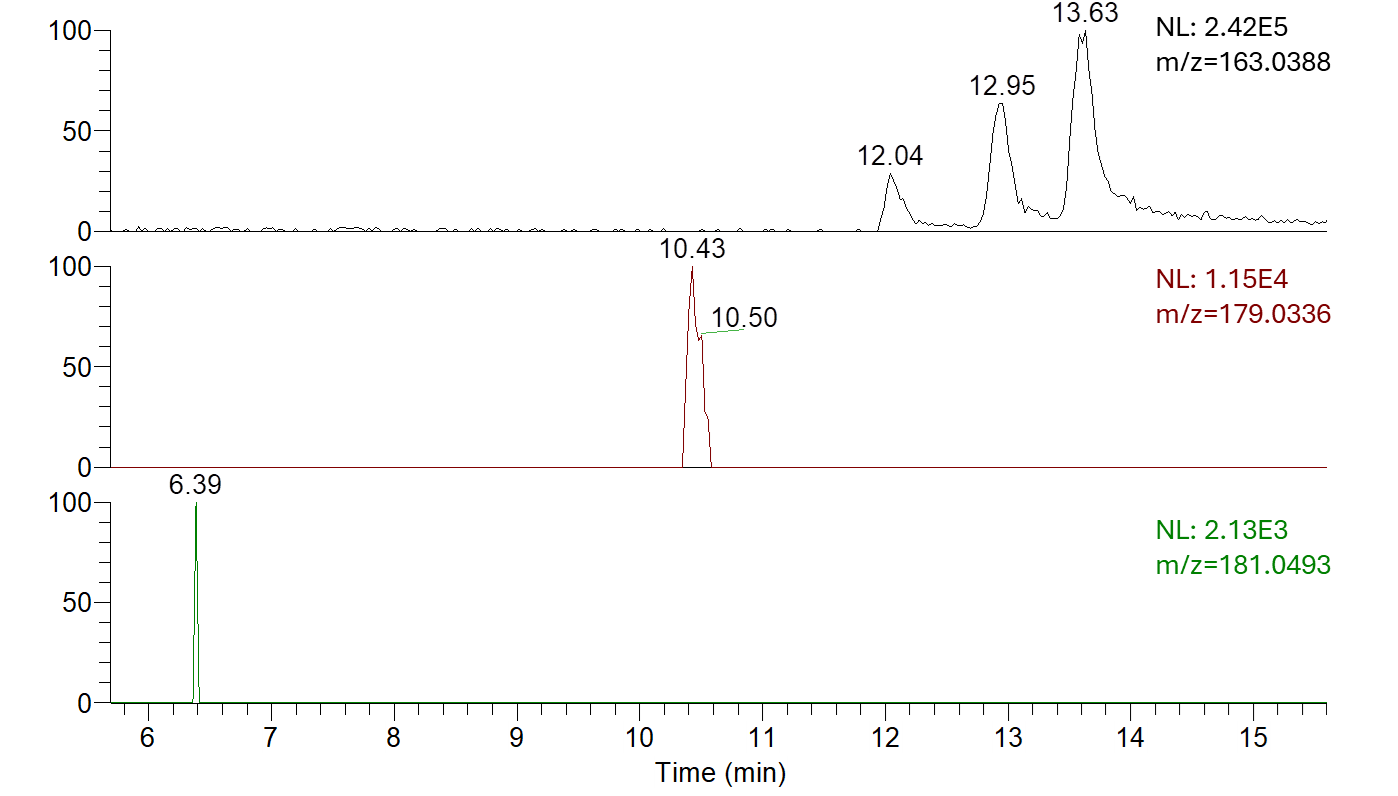


**Figure S10.** Chromatographic separation of transformation products formed from coumarin. NL, normalized scale.


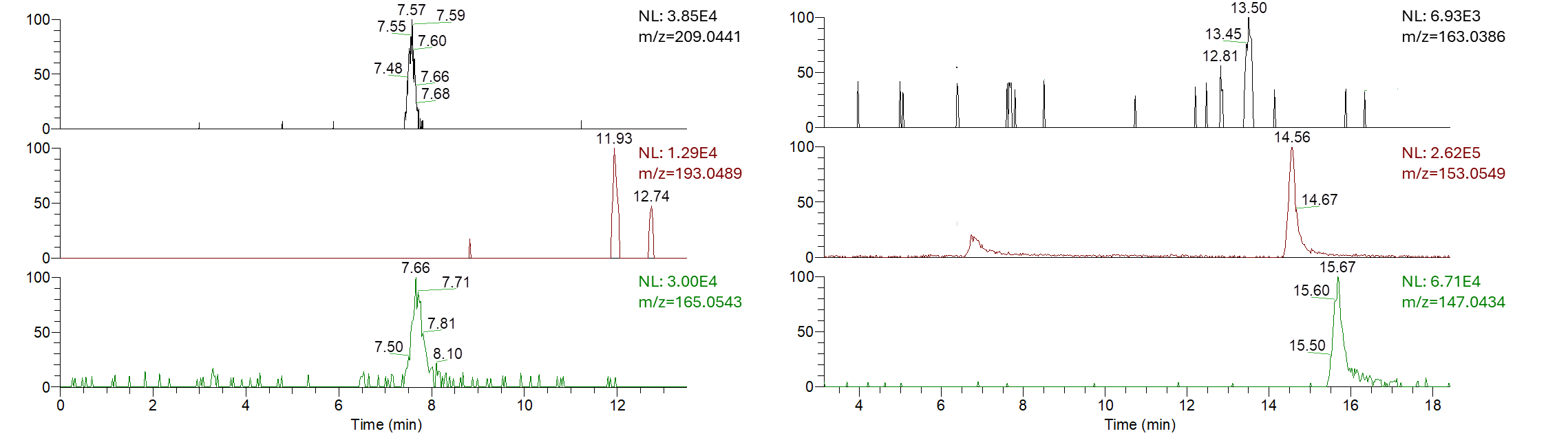


**Figure S11.** Chromatographic separation of transformation products formed from hymecromone. NL, normalized scale.


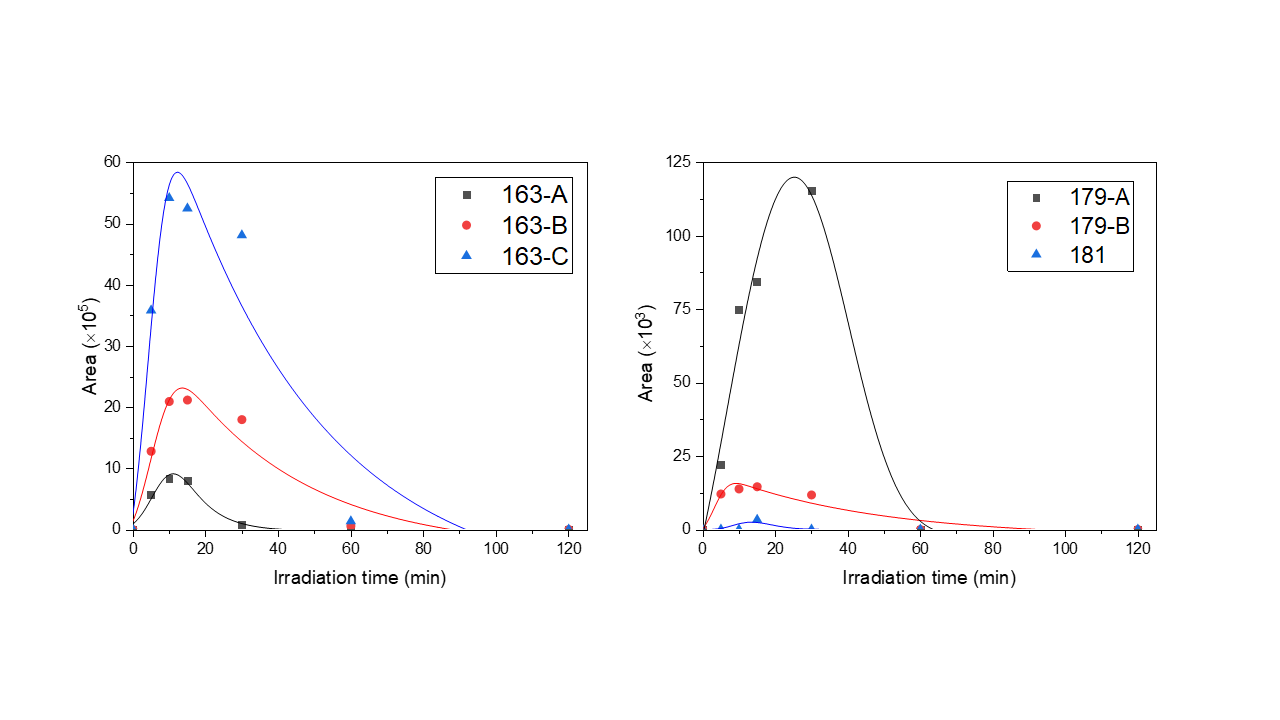


**Figure S12.** Transformation products indicated as [MH]^+^ formed from coumarin degradation as a function of the UV-A light irradiation time in the presence of 400 ppm TiO_2_.


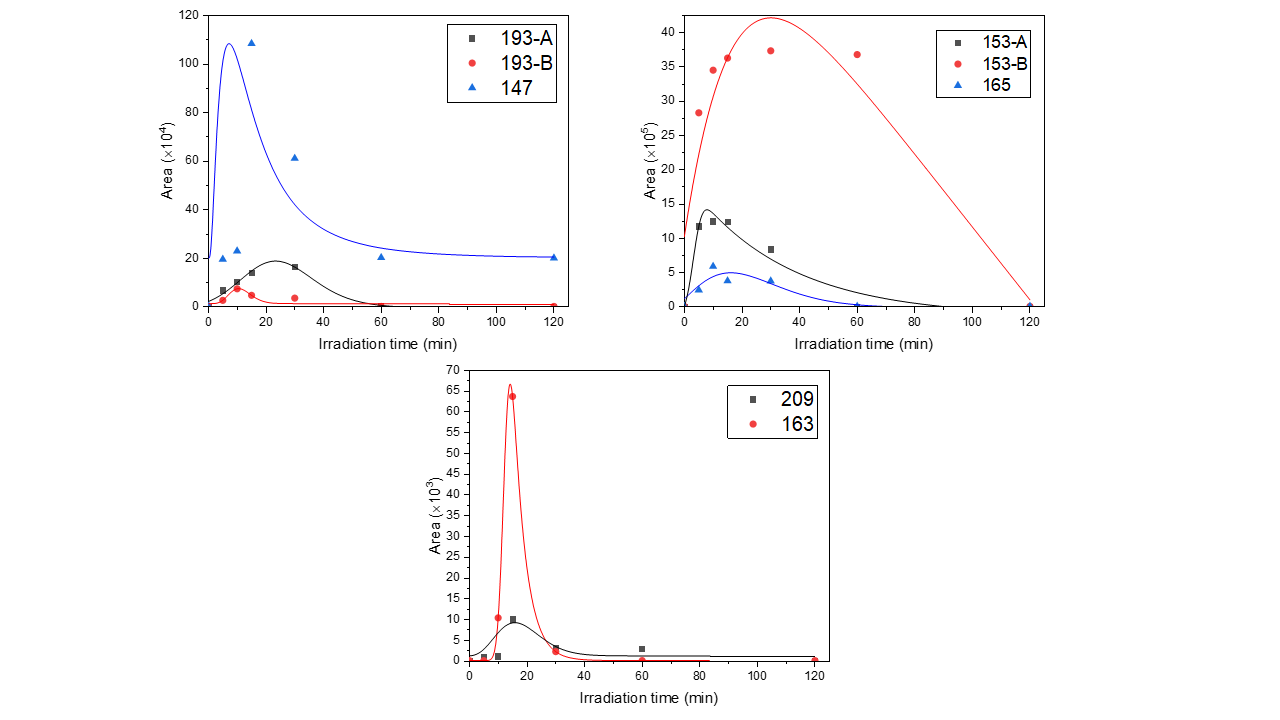


**Figure S13.** Transformation products indicated as [MH]^+^ formed from hymecromone degradation as a function of the UV-A light irradiation time in the presence of 400 ppm TiO_2_.

**Table S6.** List of [MH]^+^ and fragments from MS^n^ spectra with their empirical formulas and Δppm of coumarin TPs.

| **[MH]^+^ (*m/z*)** | **Empirical formula of [MH]^+^** | **MW** | **t_R_ (min)** | **MS^2^ (%)** | **Δppm** | **MS^3^ (%)** | **Δppm** |
| --- | --- | --- | --- | --- | --- | --- | --- |
| 163.0387  163-A | C_9_H_7_O_3_ | 162-A | 11.98 | 103.0549 C_8_H_7_ [100] (-CO_2_) | 6.144 | - | - |
|  |  |  |  | 91.0547 C_7_H_7_ [1] (-2CO) | 5.087 | - | - |
|  |  |  |  | 107.0493 C_7_H_7_O [39] (-2CO) | 1.108 | - | - |
|  |  |  |  | 91.0543 C_7_H_7_ [29] (-CO,-CO_2_) | 0.914 | - | - |
| 163.0386  163-B |  | 162-B | 12.87 | 135.0442 C_8_H_7_O_2_ [6] (-CO) | 0.844 | 107.0493 C_7_H_7_O [100] (-CO) | -3.641 |
|  |  |  |  | 119.0492 C_8_H_7_O [100] (-CO_2_) | 0.912 | 91.0543 C_7_H_7_ [100] (-CO) | -3.681 |
|  |  |  |  | 107.0492 C_7_H_7_O [17] (-2CO) | 1.295 | - | - |
|  |  |  |  | 91.0543 C_7_H_7_ [32] (-CO. -CO_2_) | 1.902 | - | - |
| 163.0388  163-C |  | 162-C | 13.52 | 135.0442 C_8_H_7_O_2_ [2] (-CO) | 1.006 | 107.0493 C_7_H_7_O [52] (-CO) | -4.389 |
|  |  |  |  | 119.0492 C_8_H_7_O [100] (-CO_2_) | 1.500 | 91.0543 C_7_H_7_ [100] (-CO) | -3.352 |
|  |  |  |  | 107.0492 C_7_H_7_O [5] (-2CO) | 2.135 | - | - |
|  |  |  |  | 91.0543 C_7_H_7_ [65] (-CO,-CO_2_) | 2.341 | - | - |
| 179.0336  179-A | C_9_H_7_O_4_ | 178-A | 10.43 | 150.0313 C_8_H_6_O_3_ [9] (-CHO) | -2.959 | - | - |
|  |  |  |  | 135.0442 C_8_H_7_O_2_ [100] (-CO_2_) | -3.069 | 107.0492 C_7_H_7_O [100] (-CO) | -4.015 |
|  |  |  |  | 133.0284 C_8_H_5_O_2_ [56] (-HCOOH) | -1.536 | 105.0337 C_7_H_5_O [36] (-CO) | -3.235 |
|  |  |  |  | 122.0363 C_7_H_6_O_2_ [36] (-C_2_HO_2_) | -3.846 | - | - |
|  |  |  |  | 117.0337 C_8_H_5_O [<1] (-CH_2_O_3_) | -2.903 | - | - |
|  |  |  |  | 107.0492 C_7_H_7_O [<1] (-CO,-CO_2_) | -4.295 | - | - |
| 179.0333  179-B |  | 178-B | 10.50 | 135.0439 C_8_H_7_O_2_ [95] (-CO_2_) | -1.143 | - | - |
|  |  |  |  | 133.0284 C_8_H_5_O_2_ [48] (-HCOOH) | -3.942 | 105.0336 C_7_H_5_O [79] (-CO) | -3.996 |
| 181.0493 | C_9_H_9_O_4_ | 180 | 6.39 | 135.0439 C_8_H_7_O_2_ [80] (-HCOOH) | -4.994 | 107.0493 C_7_H_7_O [100] (-CO) | -5.997 |
|  |  |  |  | 107.0492 C_7_H_7_O [100] (-C_2_H_2_O_3_) | -4.669 | - | - |

**Table S7.** List of [MH]^+^ and fragments from MS^n^ spectra with their empirical formulas and Δppm of hymecromone TPs.

| **[MH]^+^ (*m/z*)** | **Empirical formula of [MH]^+^** | **MW** | **t_R_ (min)** | **MS^2^ (%)** | **Δppm** | **MS^3^ (%)** | **Δppm** |
| --- | --- | --- | --- | --- | --- | --- | --- |
| 193.0482  193-A | C_10_H_9_O_4_ | 192-A | 12.02 | 165.0546 C_9_H_9_O_3_ [12] (-CO) | 0.066 | 147.0439 C_9_H_7_O_2_ [100] (-H_2_O) | -4.994 |
|  |  |  |  | 147.0441 C_9_H_7_O_2_ [47] (-HCOOH) | 0.786 | 119.0492 C_8_H_7_O [100] (-CO) | -4.030 |
|  |  |  |  | 137.0604 C_8_H_9_O_2_ [22] (-2CO) | 1.135 | - | - |
|  |  |  |  | 131.0491 C_9_H_7_O [8] (-CH_2_O_3_) | 1.144 | - | - |
|  |  |  |  | 123.0441 C_7_H_7_O_2_ [11] (-C_3_H_2_O_2_) | 0.939 | - | - |
|  |  |  |  | 103.0548 C_8_H_7_ [68] (-C_2_H_2_O_4_) | -0.148 | - | - |
| 193.0482  193-B |  | 192-B | 12.82 | 147.0447 C_9_H_7_O_2_ [43] (-HCOOH) | 0.990 | 119.0497 C_8_H_7_O [100] (-CO) | -5.206 |
|  |  |  |  | 119.0497 C_8_H_7_O [32] (-C_2_H_2_O_3_) | 0.254 | - | - |
|  |  |  |  | 103.0548 C_8_H_7_ [31] (-C_2_H_2_O_4_) | 0.144 | - | - |
| 209.0441 | C_10_H_9_O_5_ | 208 | 7.57 | 191.0336 C_10_H_7_O_4_ [100] (-H_2_O) | -1.388 | 163.0387 C_9_H_7_O_3_ [100] (-CO) | -1.782 |
|  |  |  |  | 165.0544 C_9_H_9_O_3_ [18] (-CO_2_) | -1.337 | - | - |
|  |  |  |  | 163.0388 C_9_H_7_O_3_ [3] (-H_2_O,-CO) | -1.230 | - | - |
|  |  |  |  | 149.0233 C_8_H_5_O_3_ [4] (-C_2_H_4_O_2_) | -0.272 | - | - |
| 153.0542  153-A | C_8_H_9_O_3_ | 152-A | 6.59 | 135.0440 C_8_H_7_O_2_ [100] (-H_2_O) | -4.179 | 107.0491 C_7_H_7_O [100] (-CO) | -5.510 |
|  |  |  |  | 111.0441 C_6_H_7_O_2_ [15] (-C_2_H_2_O) | -3.732 | - | - |
|  |  |  |  | 107.0491 C_7_H_7_O [2] (-HCOOH) | -5.136 | - | - |
| 153.0542  153-B |  | 152-B | 14.55 | 135.0441 C_8_H_7_O_2_ [100] (-H_2_O) | -4.328 | 107.0492 C_7_H_7_O [100] (-CO) | -5.136 |
|  |  |  |  | 111.0441 C_6_H_7_O_2_ [12] (-C_2_H_2_O) | -4.452 | - | - |
|  |  |  |  | 107.0492 C_7_H_7_O [3] (-HCOOH) | -4.202 | - | - |
| 163.0384 | C_9_H_7_O_3_ | 162 | 13.50 | 119.0492 C_8_H_7_O [100] (-CO_2_) | -4.030 | - | - |
|  |  |  |  | 91.0543 C_7_H_7_ [42] (-CO,-CO_2_) | -5.439 | - | - |
| 147.0437 | C_9_H_7_O_2_ | 146 | 15.67 | 103.0543 C_8_H_7_ [100] (-CO_2_) | -4.805 | - | - |
|  |  |  |  | 91.0542 C_7_H_7_ [42] (-2CO) | -6.207 | - | - |
| 165.0543 | C_9_H_9_O_3_ | 164 | 7.66 | 137.0597 C_8_H_9_O_2_ [19] (-CO) | -3.900 | - | - |
|  |  |  |  | 119.0493 C_8_H_7_O [100] (-HCOOH) | -3.610 | 91.0542 C_7_H_7_ [100] (-CO) | -5.439 |


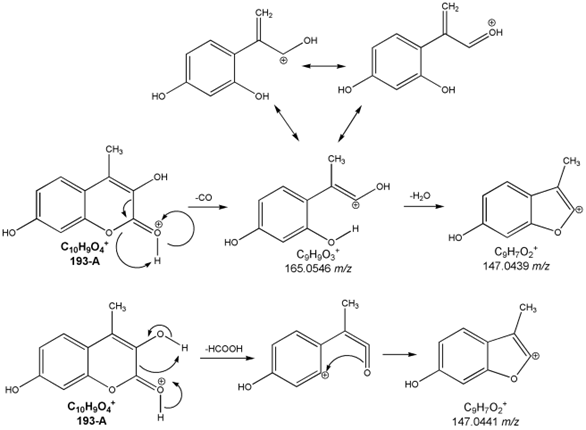


**Figure S14.** Proposed formation mechanism of 165.0546 *m/z* with its product ion at 147.0440 *m/z*, and 147.0440 *m/z* from intermediate 193-A.


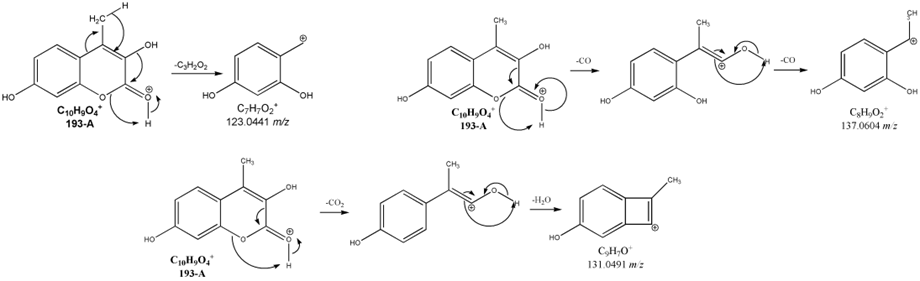


**Figure S15.** Proposed formation mechanism of 123.0441 *m/z*, 137.0604 *m/z* and 131.0491 *m/z* from 193-A.


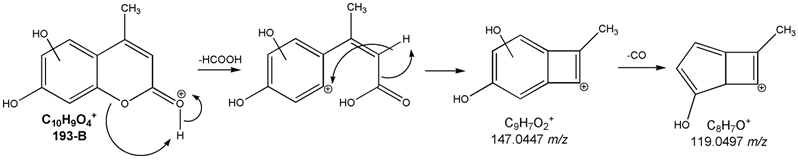


**Figure S16.** Proposed formation mechanism of 147.0447 *m/z* with its product ion at 119.0497 *m/z* from 193-B.


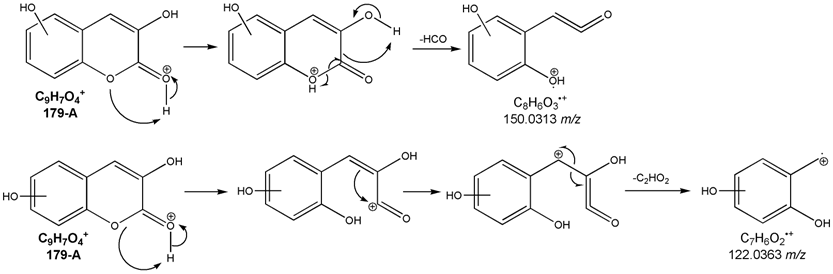


**Figure S17.** Proposed formation mechanism of 150.0313 *m/z* and 122.0363 *m/z* from 179-A.


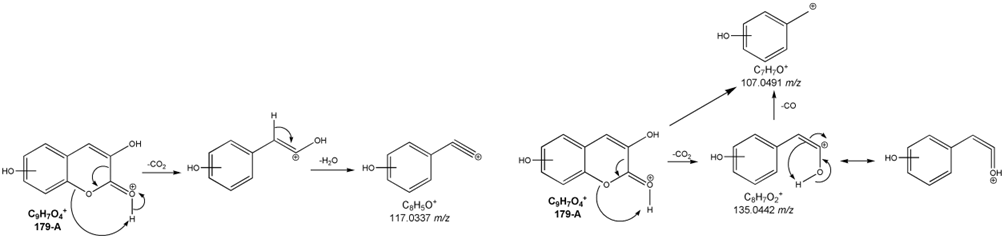


**Figure S18.** Proposed formation mechanism of 117.0337 *m/z* and 135.0442 *m/z* with its product ion at 107.0491 *m/z* from 179-A.
